# Evidence for intermittent coupling of intramyocardial small, engineered heart tissues acutely implanted into rabbit myocardium

**DOI:** 10.1093/cvr/cvaf034

**Published:** 2025-02-28

**Authors:** Eline Huethorst, Martin J Bishop, Francis L Burton, Chris Denning, Nikolaj Gadegaard, Rachel C Myles, Godfrey L Smith

**Affiliations:** School of Cardiovascular and Metabolic Health, University of Glasgow, Glasgow G12 8TD, UK; Research Department for Digital Twins in Healthcare, School of Biomedical Engineering and Imaging Sciences, King’s College London, London SE1 7AR, UK; School of Cardiovascular and Metabolic Health, University of Glasgow, Glasgow G12 8TD, UK; Biodiscovery Institute, Faculty of Medicine and Health Sciences, University of Nottingham, Nottingham NG7 2RD, UK; School of Engineering, University of Glasgow, Glasgow G12 8LT, UK; School of Cardiovascular and Metabolic Health, University of Glasgow, Glasgow G12 8TD, UK; School of Cardiovascular and Metabolic Health, University of Glasgow, Glasgow G12 8TD, UK

**Keywords:** HiPSC-CM implantation, Graft-host coupling, Intramyocardial implantation, Rabbit heart, Electric field effect

## Abstract

**Aims:**

Electrical integration of human-induced pluripotent stem-cell-derived cardiomyocyte (hiPSC-CM)-based tissue with the host myocardium is a requirement of successful regeneration therapy. This study was designed to identify electrical coupling in the acute phase (1–2 h) post-grafting using an *ex vivo* model.

**Methods and results:**

Small, engineered heart tissues (mini-EHTs), consisting of ∼50 000 hiPSC-CMs on a hydrogel (spontaneous rate 0.34 ± 0.05 Hz), were loaded with Cal520-AM. EHTs were implanted sub-epicardially into a Langendorff-perfused rabbit heart after blebbistatin treatment. For up to 100 min, a continuous pseudo-electrocardiogram was recorded during sinus rhythm (rate 2.0–3.5 Hz). At 25 min intervals, EHT calcium transients (CaTs) were recorded for 10–20 s (no contraction group). To study the influence of mechanical activity, blebbistatin was washed off after implantation (contraction recovery group). Periodic entrainment of EHTs with the myocardium was detected less often (*P* = 0.011) in the no contraction group (1/9 hearts) than in the contraction recovery group (5/6 hearts). The average coupling delay (QRS-CaT) and the difference in consecutive delays (Δdelay) were 89 ± 50 and 10 ± 3 ms, respectively (*n* = 12 traces; *N* = 6 hearts). Coupling ratios (QRS:CaT) varied from 2:1 to 4:1. These coupling parameters were not significantly different in the two experimental groups. Modelling of hiPSC-CM tissue separated by a 25 μm saline gap from the myocardium demonstrated field-effect coupling with similarly variable activation delays. Importantly, coupling failed with a gap of 100 μm.

**Conclusion:**

EHT entrainment is possible immediately after grafting and has features compatible with field-effect coupling. Sensitivity to the gap dimensions may explain why entrainment is more common in actively contracting myocardium.

## Graphical Abstract

**Graphical Abstract:**
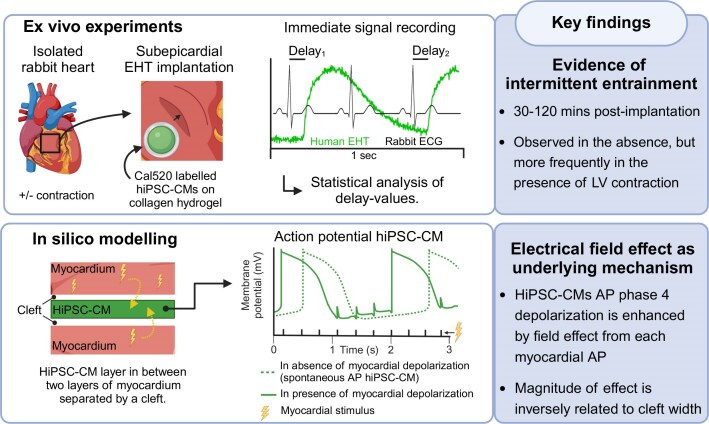

**Time for primary review: 34 days**

## 1. Introduction

Human-induced pluripotent stem-cell-derived cardiomyocytes (hiPSC-CMs) are a promising material for cardiac regeneration therapies.^1–3^ Implantation of these cells into the heart after a myocardial infarction could help repair the heart and prevent heart failure.^4,5^ To achieve mechanical benefit, these therapies rely on the electrical integration of the implanted cells so that the newly grafted myocardium contracts synchronously. The earliest high-fidelity electrical coupling observed experimentally involved the implantation of human embryonic stem-cell-derived cardiomyocytes into the guinea-pig myocardium^6^ and non-human primates,^7^ where 1:1 coupling was reported after 14 days. In other studies, 1:1 or >2:1 coupling was observed at later time points, e.g. 4–12 weeks post-implantation,^8–10^ whereas, in some cases, no electrical coupling was seen over a similar timeframe.^11–13^ The format of the hiPSC-CMs may be important, and the implantation of cells or microtissues into the myocardium appears more successful at establishing coupling than cardiac patches engrafted onto the epicardium.^8,14,15^

Until sufficient cell–cell connections are established, hearts after cardiac regeneration therapy are vulnerable to ventricular arrhythmias.^7,16,17^ These are potentially caused by incomplete electrical coupling and/or the electrophysiological mismatch between the adult host myocardium and immature hiPSC-CMs within the graft.^18^ Various *in vivo* studies have reported that most arrhythmias occur during the first 2–4 weeks post-implantation^7,15–17,19^ and sometimes over a longer period of time.^9^ The role of the newly introduced myocytes in the arrhythmia is unclear. Liu *et al*.^17^ could not identify a re-entrant pathway, and instead hypothesized that the arrhythmia originated from a focal source in the graft region, a conclusion supported by others.^16^ Chong *et al.*^7^ compared their study in non-human primates to previous studies in smaller animal models and suggested that the larger graft size and lower heart rate might be the cause of the arrhythmias seen. In none of these studies were grafts studied within hours after implantation.

It is unclear how quickly implanted cells can establish electrical coupling with the host myocardium, it is also not known whether ventricular contraction is a positive or negative factor.^20^ Early studies using chick cardiomyocyte aggregates showed exponentially increasing coupling efficiency with a half-time of 30 min.^21^ Others have shown that neonatal rat cardiomyocytes can form gap–junction (GJ) coupling within 5–15 min of two cells being in close (<1 µm) contact.^22,23^ Other *in vitro* studies have shown evidence of electrical coupling between two closely apposed hiPSC-CM layers within 2 h;^24^ therefore, the formation of electrical coupling between host and implanted myocardium over the initial 1–2 h appears feasible.

This study was performed in healthy hearts (as opposed to infarct scars) in order to provide a consistent environment that maximized the chances of coupling for this initial examination of host–graft interactions shortly after implantation (<2 h). Small, engineered heart tissues (mini-EHTs), made of a syncytium of hiPSC-CMs seeded on a hydrogel, were grafted into the left ventricular (LV) free wall of healthy isolated rabbit hearts. Since ventricular contraction may trigger entrainment through a variety of mechano-electrical pathways, entrainment was examined in the absence and presence of ventricular contraction. Rabbit hearts were used for this study because they have a similar cardiac electrophysiology and arrhythmia dynamics to that of humans, and the range of basal heart rates in rabbits overlaps with the highest rates achieved in human hearts.^25–27^ A commercial hiPSC-CM line was used for the following reasons: (i) commercial cell lines go through quality control processes to minimize batch-to-batch variation and, therefore, represent a convenient and reliable experimental material;^28^ (ii) commercial cell lines are in the public domain with background literature on aspects of their physiology/electrophysiology, including a published and validated *in silico* model of cellular electrophysiology;^29^ (iii) using a readily available commercial line allows this work to be replicated and further developed by the scientific community.

Electrical interactions between healthy normal myocardium and EHTs were studied by monitoring calcium transient (CaT) signals from the EHTs alongside pseudo-electrocardiograms (pECGs) from the heart, and using circular statistics to test for the presence of entrainment.^30,31^ The analysis revealed that there was periodic coupling between the host myocardium and the EHTs occurring as early as 30 min after implantation. Periodic coupling was more commonly observed in contracting compared with non-contracting myocardium. Computational modelling indicated that transient electric fields generated by the host myocardium inside the cleft containing the EHT are sufficient to depolarize tissue and therefore steepen the pacemaker potential (Phase 4) of the EHT and promote spontaneous action potentials (APs). Therefore, field effects across the engrafted EHT rather than GJs may be important in promoting the entrainment of the electrical activity during the acute phase of engraftment between an EHT and host myocardium.

## 2. Methods

### 2.1 Animal ethics

New Zealand White rabbits (*N* = 15 rabbits, male, 2.5–3.5 kg) were used for these studies. All animal experiments were approved by the British Council for Animal Research and were conducted in accordance with the UK Animals (Scientific Procedures) Act 1986 and guidelines from Directive 2010/63/EU under Project Licence (PP5254544). All animals were kept and treated in compliance with the local regulations for animal welfare. All isolated heart experiments were performed in accordance with animal welfare guidelines.

### 2.2 Preparation of a hydrogel substrate

Freeze-dried methacrylated recombinant collagen–like peptide [RCP-MA, Fujifilm Cellular Dynamics International (FCDI, Madison, WI)] was dissolved in PBS^+/+^ (10% w/v) and placed in a 37°C water bath for 1 h. Lithium phenyl(24,6-trimethylbenzoyl) phosphinate (LAP, L0290, Tokyo Chemical Industry UK Ltd, Oxford, UK) was used as a crosslinking agent and dissolved in PBS (2.5% w/w), followed by a 30 min incubation at 37°C. Then, RCP-MA, LAP, and fibronectin (FN, bovine, F1141, Sigma Aldrich, Gillingham, SP8 4XT) were mixed in a ratio of 1000:26:50 µL. The mixture was pipetted into a 6 mm diameter circular mould of roughly 350 μm thick and covered with a coverslip, preventing any air gaps. Samples were exposed to 365 nm UV light at a light intensity of 10 mW/cm^2^ for 5 min using the Omnicure series 1500 crosslinker, resulting in hydrogels with a Young’s modulus of 22.6 kPa (FujiFilm data). After crosslinking, the hydrogels were placed into a 10% mixture of FN with PBS^+/+^ over night at 4°C.^32^

### 2.3 HiPSC-CM culture and EHT development

HiPSC-CMs, (FCDI, ICell^2^, 01434) were stored in liquid nitrogen upon receipt and cultured as per guidance from the manufacturer. Briefly, RCP-MA hydrogels were placed onto a glass-bottom well plate each (MatTek, Bratislava, Slovak Republik) and incubated with plating medium at room temperature. HiPSC-CMs were thawed, diluted with plating medium, and counted using trypan blue and a haemocytometer. Cells were centrifuged for 5 min at 300 *g* after which the supernatant was discarded, and the cells were resuspended in CDI culture medium to get a concentration of 1.5 × 10^6^ cells/mL. Medium was taken off the hydrogels, leaving the hydrogel only, where a silicone stencil with an inner diameter of 3 mm was placed on top of the hydrogel. Then, using a long, thin pipette tip, 30 µL of the cell suspension was seeded inside the stencil and on top of the hydrogel (∼45 000 cells). The stencil was removed on Day 2 after cell seeding. Culture medium was changed every 3–4 days.

### 2.4 *In vitro* excitation–contraction–coupling measurements

One day before measurements, EHT maintenance medium was changed to serum-free and phenol-red-free medium (BMCC). On the day of measurements, EHTs were incubated with Cal590-AM (1 μM, 20511, AAT Bioquest, Pleasanton, CA), FluoVolt (1:1000, F10488, ThermoFisher, Waltham, MA), and 0.02% pluronic acid for 30 min and washed 2× with BMCC medium. Recordings were made on a CellOPTIQ *in vitro* platform that allows for simultaneous recording of voltage, intracellular Ca^2+^ and contractility in suitably treated preparations. This platform includes an onstage incubator that maintains a temperature of 37°C, 85% humidity, and 5% CO_2_. For this set of experiments, a 40× objective (Olympus, Tokyo, Japan, NA = 0.6) was used. The optical setup consisted of a 580/25 nm light-emitting diode (LED) and a 470/40 nm LED, which excite the Cal590-AM and FluoVolt dyes, respectively. PMT1 collects all <550 nm light (FluoVolt, AP), whereas PMT2 collects all light of 645/75 nm (Cal590, CaT). Transients were recorded simultaneously by automatically alternating the respective LEDs on/off with 1 ms intervals, so that only one LED emits light at a time. White light images were recorded with a digital camera at 100 fps (Hamamatsu ORCA-flash 4.0 V2 digital CMOS camera C11440-22CU) to reconstruct videos of contracting CMs. Dichroic mirrors ensure the light of the correct wavelengths reaches the EHT, and emitted fluorescent light is directed to the PMT or high-speed camera. APs and CaTs were analysed with in-house software. Contraction was analysed with the open-access contractility algorithm MUSCLEMOTION.^33^ HiPSC-CMs were electrically stimulated using graphite electrodes at various pacing frequencies (1, 2, 2.5, 2.9, and 3.3 Hz). Capture rates were analysed, and preparations were classified as one of three categories: follow, alternans, or drop-out. Alternans were defined as alternating AP duration or CaT amplitude of >10%. Cells not able to follow the stimulus at an AP:stimulus ratio of 1:1 were classified as drop-out.

### 2.5 Rabbit anaesthesia and heart isolation

Rabbits (*N* = 15) were anaesthetized with a single intravenous injection of 0.5 mL/kg Euthatal (200 mg/mL, sodium pentobarbitone, Rhoe Merieux Inc., Athens, GA, USA) mixed with 500 U of heparin (CP Pharmaceutical Ltd, Wrexham, UK) via the left marginal ear vein. After confirming adequate anaesthesia by testing for the absence of any pain reflex, hearts were excised and placed in ice-cold heparin-substituted Tyrode’s solution (92.3 mM NaCl; 20 mM NaHCO_3_; 1 mM Na_2_HPO_4_; 1 mM MgSO_4_; 5 mM KCl; 20 mM sodium acetate; 25 mM glucose; 1.8 mM CaCl_2_; 5 mM HEPES). Then, hearts were mounted on a Langendorff perfusion rig and perfused with warm (37°C) Tyrode’s solution at a constant flow of 40 mL/min. A balloon was inserted inside the LV for continuous measurements of dynamic ventricular pressure. Subsequently, hearts were incubated with 10 µM blebbistatin for 20 min to uncouple contraction. The heart was supported by a cradle containing two Ag/AgCl electrodes that were in proximity to the right ventricular/LV surface, resulting in a recorded voltage signal showing QRS and T complexes that represent a pECG.

### 2.6 Simultaneous recordings of rabbit heart pECG and remote APs

Dye solution was prepared in 100 mL of Tyrode’s solution containing 10 μM blebbistatin, 25 μL FluoVolt, and 0.1% pluronic acid. Perfusion rate of the rabbit heart (*N* = 8) was slowed down to 26 mL/min, whereafter the dye solution was perfused through the heart. The effluent of the heart was collected, filtered using a 0.22 μm filter, and re-perfused for 20 min in total. A fibreoptic light guide together with LEDs and PMTs was used to record APs, with signals recorded using LabChart (v8.0, AdInstruments, Oxford, UK).

### 2.7 Simultaneous pECG and EHT CaT measurements in *ex vivo* hearts

Spontaneously, beating EHTs were implanted on Days 9–11 after cell seeding. Prior to implantation, EHTs were stained with 2 µM Cal520-AM (21130, AAT Bioquest, Pleasanton, CA) in the presence of 0.02% pluronic acid for 1 h, subsequently washed twice with BMCC medium and placed back in the incubator to allow for maximal cleavage of the -AM group of the intracellular ester form of the dye. Prior to implantation, CaTs were recorded from EHTs *in vitro* as a quality control for the intrinsic rate (IR) and the CaT signal strength. Cal520-AM was chosen for its brightness compared with Cal590-AM (see Supplementary material online, *S1*), but due to its comparable wavelength to FluoVolt, dual imaging of rabbit myocardial APs from the incision site using FluoVolt was not possible. Therefore, calcium traces from the EHT were compared with the recorded pECG.

**Figure 1:**
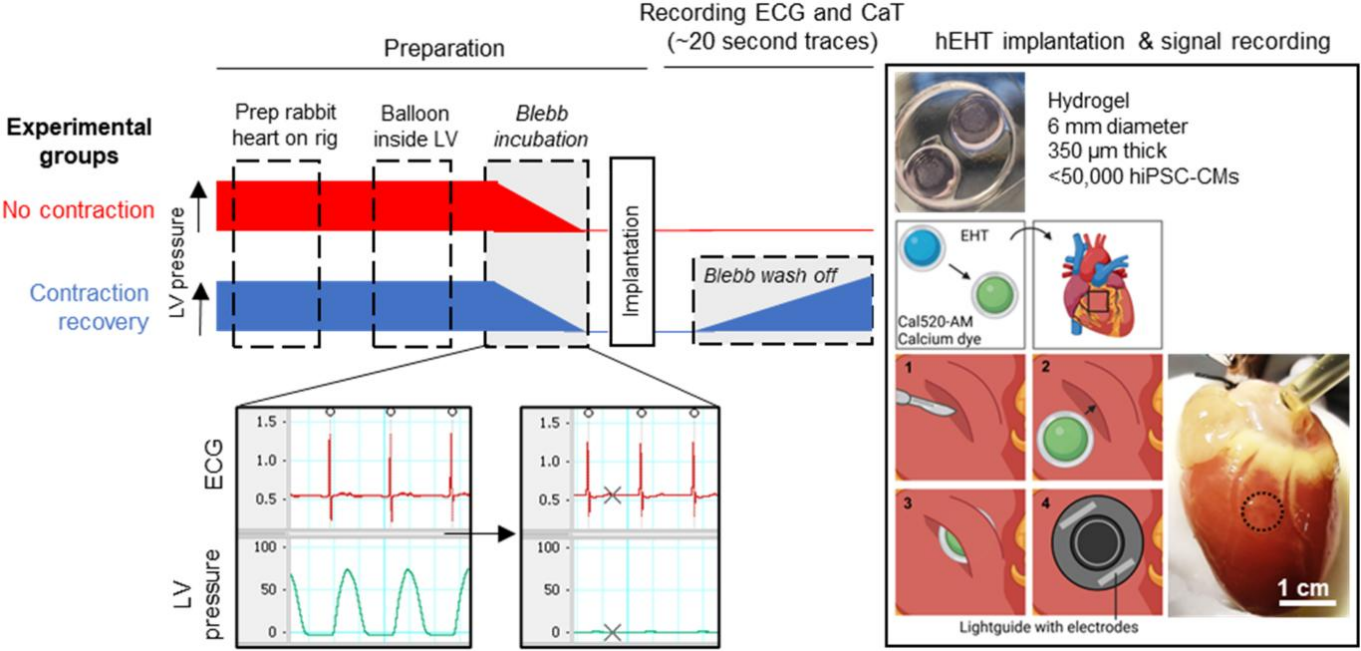
Experimental setup: hearts are placed on the Langendorff perfusion rig, perfused with Tyrode’s solution, and monitored using continuous ECG recordings, after which a balloon is inserted into the LV to measure LV pressure. Hearts are then exposed to one of two protocols: (i) ‘No Contraction’, where the contraction is uncoupled using blebbistatin (blebb) for the whole duration of the experiment (top bar), and (ii) ‘Contraction Recovery’, where the contraction is uncoupled using blebbistatin for a small period of time, after which the blebbistatin is washed off and contraction recovered (bottom bar). This is illustrated by the thickness of both bars. Right-handed box: human EHTs are stained with Cal520-AM and implanted into the myocardium by creating a small incision underneath the epicardium and placing the human EHT inside the pocket (Boxes 1–3). To record Cal520 signals, a light guide is placed on top of the implantation site (Box 4), which contains electrodes to field stimulate the implanted cells. An example photograph of a heart with an implanted EHT is shown on the right-hand side. The scale bar indicates 1 cm. After implantation, the first Cal520 recording is made (*T* = 0 min, start), after which the blebbistatin is washed of in the gradual contraction group. Further recordings are made every 20–30 min. Created in BioRender. Huethorst, E. (2025) https://BioRender.com/j30o578.

Cal520-loaded EHTs were implanted intramyocardially by making a superficial incision underneath the epicardium (see Supplementary material online, *Figure S2A*), creating a pocket into which the EHT was inserted so that it was sandwiched between the upper epicardial flap and the underlying myocardium (*Figure 1*). A light guide with pacing electrodes on either side of the aperture was placed over the implantation site to record CaTs from the EHTs. After implantation, most EHTs (*N* = 10/15) showed spontaneous CaT signals directly post-implantation (i.e. *T* = 0). However, for some EHTs (*N* = 5/15), spontaneous CaTs were only seen 20–30 min later. EHTs that did not show a clear calcium signal after the initial 30 min were excluded from this study.

Hearts were divided into two groups: (i) no contraction (*N* = 9 hearts), where blebbistatin was maintained in the perfusion solution and (ii) contraction recovery (*N* = 6 hearts), where blebbistatin was removed from the perfusion medium immediately after the EHT was implanted. CaTs were recorded every 20–30 min for at least 1.5 h, where each recording sequence would start with the spontaneous rate, followed by local pacing at various pacing frequencies (1, 2, 2.5, 2.9, and 3.3 Hz) to check hiPSC-CM viability. Here, each separate recording lasted 10–20 s. The first recording was immediately after implantation (0 min); thereafter, blebbistatin was washed off in the contraction recovery group only. Hearts in the no contraction group were exposed to blebbistatin for the duration of the experiment, and thus, no contraction was recorded at any time point. ECG was recorded continuously alongside the calcium traces and LV pressure using LabChart. Recording sampling rates were 1 and 10 kHz for the CaT and ECG, respectively. An overview of the experimental timeline is shown in *Figure 1*. After the experiment, hearts were prepared for histology (Supplementary material online, *Methods*) to examine the location of the hiPSC-CMs and the size of the myocardial band over the EHT (see Supplementary material online, *S2*).

### 2.8 ECG and calcium event selection

Using LabChart, CaTs were corrected for background noise and movement artefacts by subtracting the intrinsic fluorescence signal at a longer waveband (645/75 nm) that had no calcium-sensitive component. Subsequently, CaT upstroke at 50% was identified, marked, and the corresponding time points were used for later calculations. Likewise, the start of the QRS complex (Q-peak) of the pECG was identified, marked, and the corresponding time points were used for later calculations.

### 2.9 Circular statistics and selection of entrained traces

To detect a significant period of entrainment of EHT CaTs with underling electrical activity of the host myocardium in the absence of electrical pacing, successive time intervals between ECG Q-waves and CaTs were examined using a circular statistics approach.^30,31^ Here, the regular RR interval of the QRS complexes occurs every 360°, and the CaTs have a delay phase that either is uniformly distributed around this circle (e.g. a variable delay, no causal connection, and thus not-entrained) or has a common mean phase value (a constant or near-constant delay phase and thus entrained).^30^ The Rayleigh *z*-test^31^ was used to detect unimodal clustering within a cycle after converting delays to phase angles in radians. If significant clustering was found, the cluster location was estimated as the mode of the delays, calculated using the half-sample mode technique.^34^ The Rayleigh *z*-test tests the null hypothesis that events are uniformly distributed in the cycle; on the basis of the calculated *P*-value (*P*_R_), the null hypothesis is accepted or rejected. Only recordings with at least 20 QRS and 5 CaT events were analysed.

Recorded traces showed sections with and without entrainment. To find the entrained sections within each trace, all traces with a significant *P*_R_ were scanned for runs of at least five successive CaTs whose delay from the preceding ECG was within a narrow band of values around the modal delay to allow the identification of records that represented a significant period (>10 s) of consistent entrainment. The mode was calculated from a range of Δdelay values and set to ±2 standard deviations (SDs; i.e. 20 ms) of the mean Δdelay.

### 2.10 A computational model of electrophysiological coupling

An idealized computational model, representing the main biophysical features of the biological system, was used to understand the conditions under which entrainment may occur. The model consisted of a cuboid of myocardial tissue, of overall dimension 5 × 5 × 5 mm, representing normal rabbit ventricular myocardium. The upper and lower halves of the tissue were separated by a thin gap (in the *z*-direction) of thickness 100 μm. In the centre of this gap, a thin layer of hiPSC-CM tissue (representing the EHT in the experiments), of thickness 50 μm (in the *z*-direction) was defined. This layer of hiPSC-CM tissue was separated from the surrounding normal myocardium (above and below it) by a thin layer of bath (thickness 25 μm on each side). Above and below the cuboid of tissue, a 1 mm layer of bath (saline) was represented. The resolution of the computational model in the myocardial tissue region was 100 μm, which was refined to 25 μm within the thin layer of hiPSC-CM and bath through the centre in order to geometrically represent this fine structure. Additional instances of the model were also considered whereby the hiPSC-CM (EHT) layer was surrounded by 100 μm of bath between it and the surrounding myocardium (as opposed to the 25 μm default).

A full bidomain representation of the electrophysiological dynamics was used. Membrane kinetics within the rabbit myocardium region were represented by the UCLA rabbit ventricular myocyte model,^35^ with hiPSC-CM kinetics represented by the Paci model.^36^ Single-cell models were paced at basic cycle lengths of 500 and 1000 ms for 50 and 100 cycles, respectively, for the rabbit and hiPSC-CM models, respectively, to reach steady state. Saved state variables were then used to initialize tissue-level simulations. Isotropic tissue was assumed with intra- and extracellular conductivities assigned to the same value of 0.1 S/m. These set of simplifying assumptions were used to generate a minimum electrophysiological model to test mechanistic hypothesis concerning the phenomenon of entrainment observed experimentally; they were not designed to accurately reproduce tissue anatomy and/or structure.

The tissue was stimulated continuously at a cycle length of 300 ms simultaneously at two points at (0, 0, 0) and (0, 0, 5) mm to initiate symmetrical propagation through the normal myocardium towards the central hiPSC-CM layer, and in a left-to-right direction (see Supplementary material online, *S3*). To assess the spontaneous activity of the hiPSC-CM cells, a second set of simulations was also conducted in which the normal myocardium was not stimulated. Transmembrane potential (*V*_m_) and extracellular potential (*ϕ*_e_) signals were examined at sites within the thin layer of hiPSC-CM tissue, as well as surrounding rabbit myocardium. Simulations were performed with the CARPentry software.^37^

### 2.11 Statistics

GraphPad Prism v10.2.2 was used for statistical analysis. The test used for each experiment is given in the corresponding figure legends. Experimental results are shown as mean ± SD. A *P*-value <0.05 was considered statistically significant. Sample numbers are shown as *N* for experiments, hearts, rabbits, or EHTs, and as *n* for recorded traces because multiple traces were recorded from one EHT.

## 3. Results

### 3.1 The frequency dependence of excitability in mini-EHTs and rabbit myocardium

**Figure 2:**
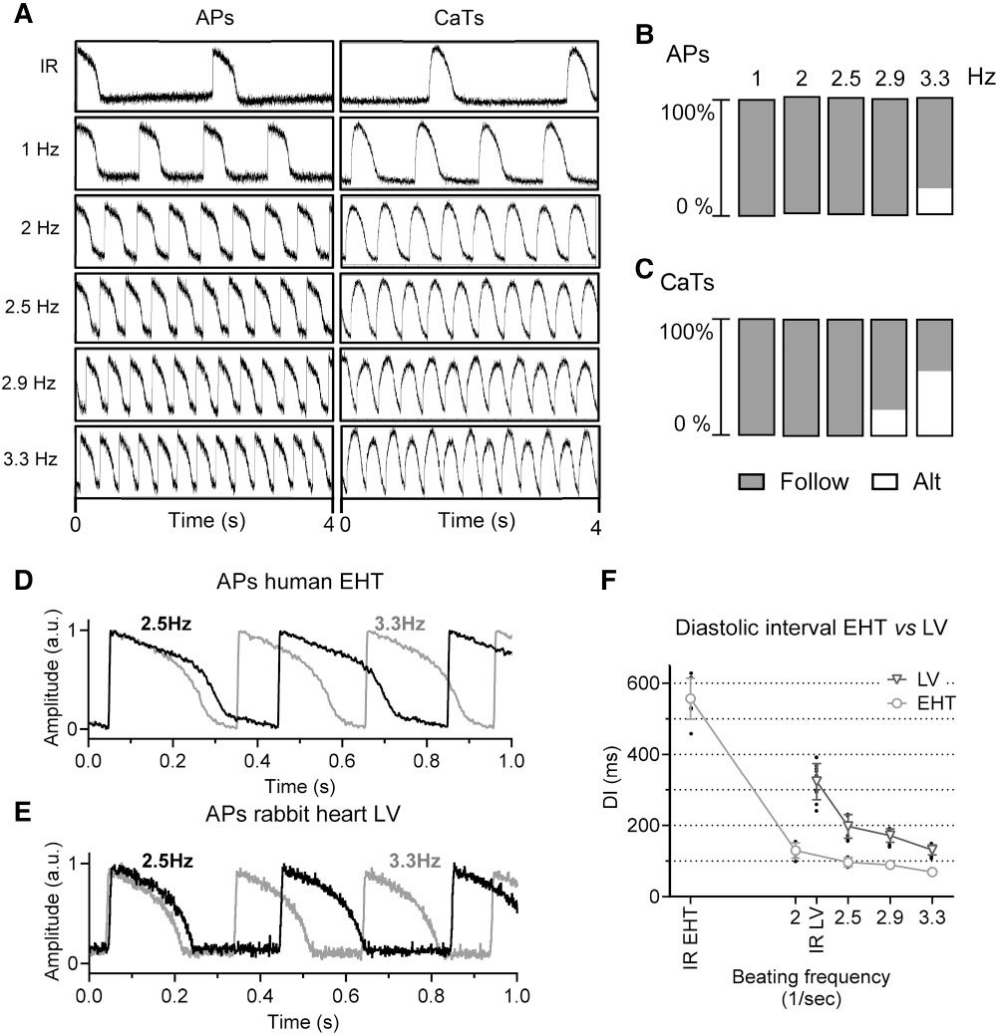
Voltage and calcium traces of EHTs subjected to electrical pacing. (*A*) Raw data traces of APs (left) and CaTs (right) at their IR and at various pacing frequencies (1, 2, 2.5, 2.9, and 3.3 Hz). (*B* and *C*) The percentage of APs (*B*) and CaT (*C*) that follow the electrical stimulus (grey), or show alternans (Alt, white) at various pacing rates (1, 2, 2.5, 2.9, and 3.3 Hz; paired data; *N* = 9 EHTs). (*D*) Example APs of the EHT at 2.5 Hz (black) and 3.3 Hz (grey). (*E*) Example APs of the rabbit LV at 2.5 Hz (black) and 3.3 Hz (grey). (*F*) Restitution curve showing the DI plotted along various beating frequencies for the rabbit LV (*N* = 8 hearts) and human EHT (*N* = 8 EHTs) during *ex vivo* and *in vitro* experiments, respectively.

The average spontaneous rate of isolated mini-EHTs used in this study was 0.34 ± 0.05 Hz (*N* = 15); this was ∼10× slower than that of the spontaneous rate of the isolated rabbit hearts (3.0 ± 0.5 Hz, *N* = 15). The capability of the mini-EHTs to achieve higher rates was assessed *in vitro* by examining the AP waveform and CaTs in preparations stained with both FluoVolt and Cal590-AM at pacing frequencies comparable with that of the isolated rabbit hearts (2–3.3 Hz). At a stimulation rate of 3.3 Hz (cycle length 300 ms), 2/9 (22%) EHTs showed APD alternans but could follow any lower pacing frequency (*Figure 2A* and *B*), indicating that a pacing cycle length of 300 ms could not be routinely supported. As shown in *Figure 2A–C*, at 2.5 Hz stimulation, no APD alternans was evident, and all EHTs responded to stimulation, but initial signs of excitation–contraction coupling failure were evident in 2/9 (22%) of the EHTs which showed CaTs with amplitude alternans of >10%. Further studies of CaTs alone (*N* = 18) show that at 2.5 Hz (400 ms cycle length), CaT alternans could be seen (2/18, 11%) and that some EHTs could not follow the faster pacing frequencies of 2.9 Hz (5/18, 28%) and 3.3 Hz (9/18, 50%), but followed a 2:1 stimulus:CaT ratio instead (data not shown). In *Figure 2D* and *E*, examples of optical APs of the rabbit LV and human EHT at two different pacing frequencies (2.5 and 3.3 Hz) demonstrate that the APD of the rabbit LV is shorter than the EHT, and therefore, the associated diastolic interval (DI) is longer. *Figure 2F* shows that on average the DI of the LV was 50–100 ms longer than that of the EHT across all cycle lengths. At a stimulation rate of 3.3 Hz, the average APD and DI of the EHTs able to respond to every stimulus were 230.9 ± 8.2 and 69.1 ± 8.2 ms (*N* = 3/7 EHTs) when compared with 167.5 ± 13.3 and 131.1 ± 13.9 ms for the rabbit LV free-wall epicardial surface (*N* = 15/15 hearts). The EHT failed to respond to higher frequencies of stimulation, while rabbit myocardium can respond to frequencies as high as 6–7 Hz.^26^ Therefore, the human mini-EHTs are unable to follow 1:1 at rabbit sinus heart rates, but stay entrained at ratios ≥2:1 robustly up to 2.5 Hz.

### 3.2 Mini-EHTs show activity following implantation

Prior to implantation, all mini-EHTs were spontaneously active *in vitro*, and their intrinsic beating rate was 0.35 ± 0.05 and 0.34 ± 0.05 Hz for the no contraction and contraction recovery groups, respectively (*P* = 0.78). The intrinsic cycle lengths of the rabbit hearts at the start of the experiment were 397 ± 57 ms (2.5 Hz) and 369 ± 58 ms (2.7 Hz) for the no contraction and contraction recovery groups, respectively [*P* = 0.36; combined: 386 ± 57 ms (2.6 Hz); range: 2–3.5 Hz].

After sub-epicardial implantation of the EHT into the rabbit heart, CaTs were recorded alongside the pECG to assess their interrelationship, both at their spontaneous rate and during a subsequent pacing sequence. Successfully implanted EHTs remained spontaneously active during the course of the experiment and did respond to electrical field stimulation. Supplementary material online, *S4* shows an overview of example traces at spontaneous and fixed rates over time. This activity was not affected by increasing LV contraction in the contraction recovery group. The averaged time course of LV pressure recovery in the contraction recovery group is shown in Supplementary material online, *S5A* (mean amplitude 32.5 ± 21.8 mmHg) and individual time points of each recording are plotted alongside the corresponding LV pressure in Supplementary material online, *S5B*. This shows that the EHT remained active with increasing LV pressure. Furthermore, histological assessment of the implantation site for Ku80, a human nuclear marker, revealed that grafted hiPSC-CMs had transferred from the hydrogel to the myocardium (see Supplementary material online, *S2B*). The histology demonstrates the overall scale of the elements, but, due to potential preservation artefacts, cannot reproduce the gaps between EHT and myocardium *in situ*. These data highlight that the viability of the EHTs was not affected during the implantation nor during the period afterwards. There were no ectopic beats in hearts with implanted EHTs in either of the experimental groups. This general behaviour indicates that the EHT did not affect the overall electrophysiology of the ventricle.

### 3.3 Irregular patterns of EHT spontaneous activity after grafting into the rabbit LV epicardium

**Figure 3:**
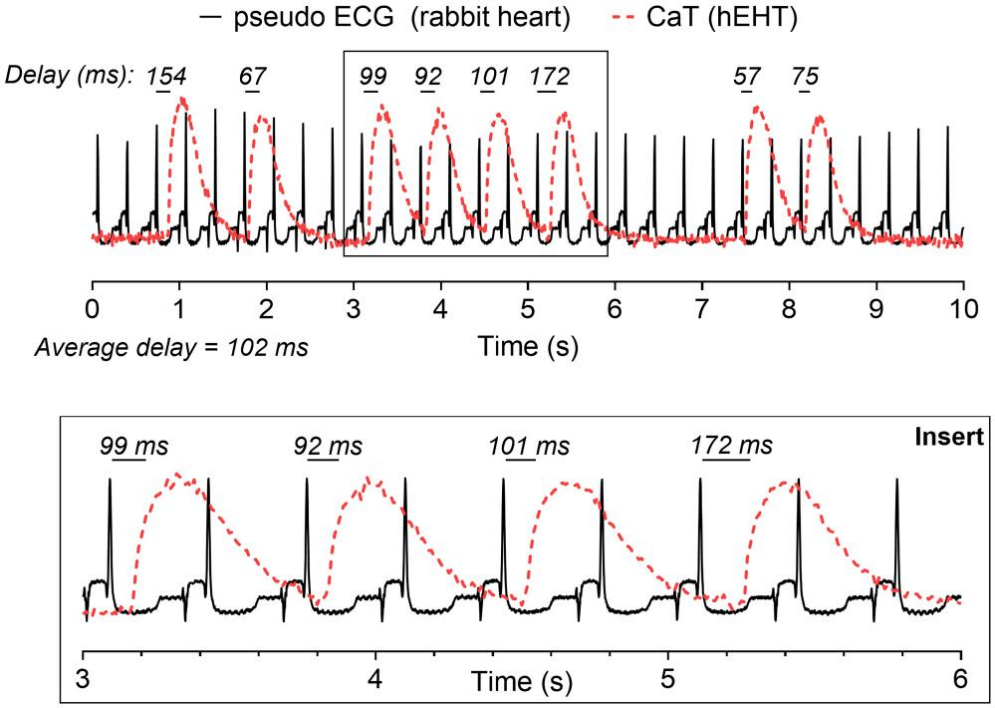
Example of a typical calcium trace (CaT; dotted, red line) alongside the pECG (continuous, black line). Top: Complete recording of a CaT and pECG at the start of the experiment. The total delay is defined as the time between the pECG and CaT upstroke and written above each time point. Here, the average total delay is 102 ms. The black box emphasizes a sequence of traces with a comparable total delay and is expanded in the insert below.

Irregular spontaneous activity of CaTs was evident after implantation, while the *in vitro* cycle length was regular. During this period, pECG-CaT delay for sequential CaT events was almost constant, suggesting that for a short period, the two events were entrained. *Figure 3* illustrates a potential intermittent phase of entrainment, where a QRS:CaT ratio of 2:1 is apparent in the middle part of the trace (insert). Additionally, the delay, which is defined as the time from the start of ventricular depolarization, i.e. the Q wave of the QRS complex, to the upstroke of the CaT, is very regular for the first three transients (average 97 ± 5 ms) but increases afterwards to 172 ms for the next complex. All records were further analysed to quantify the temporal correlation between ventricular excitation (QRS) and that of the EHT as indicated from the upstroke of the CaT. In the subsequent analysis of the extent and degree of entrainment, this particular trace was eliminated because of the brevity of the potential coupling (<5 consecutive pECG-CaT complexes with a constant delay).

### 3.4 Circular statistical assessment shows temporal correlation between myocardial and EHT activity

**Figure 4:**
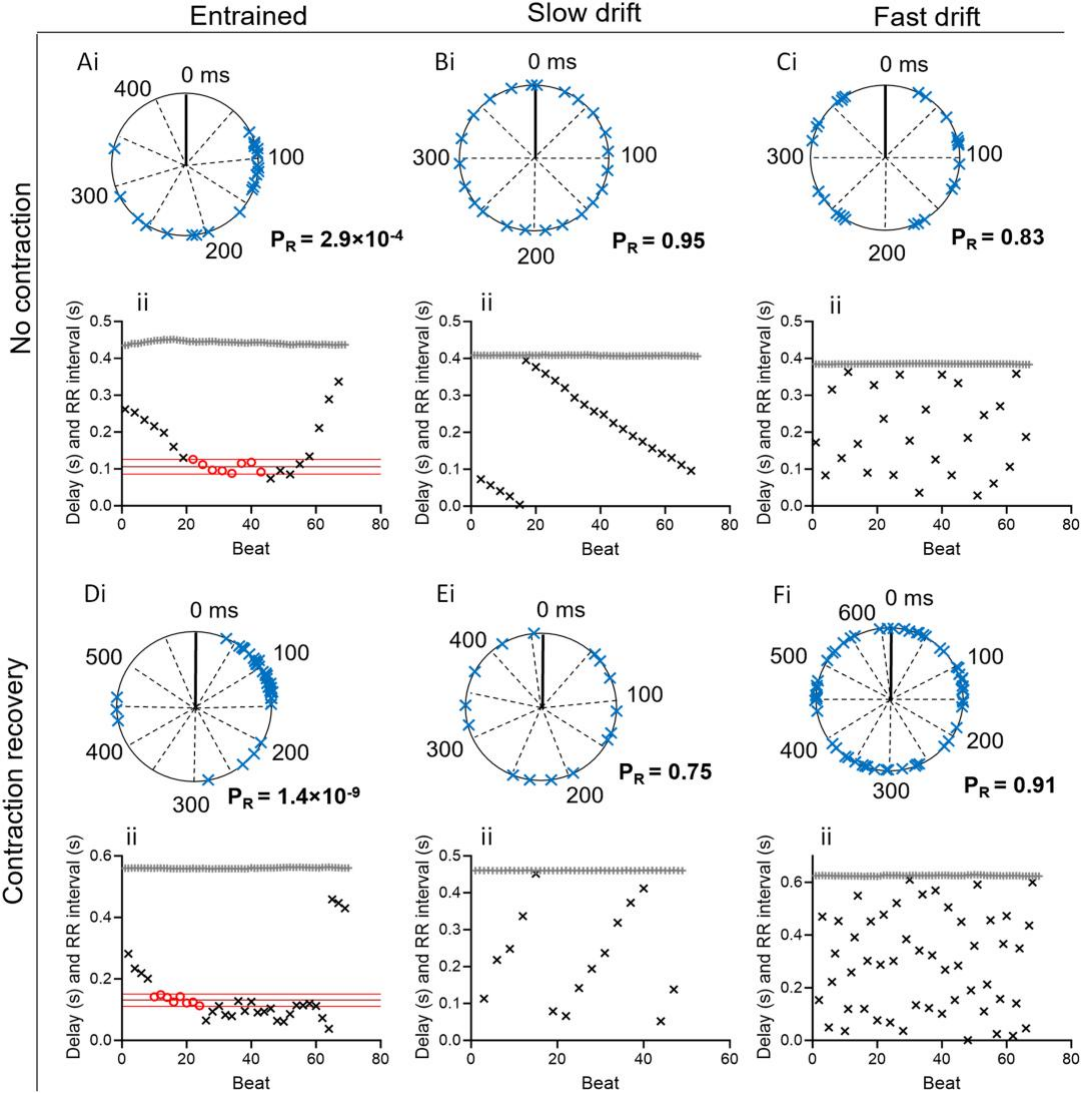
Patterns of entrained and not-entrained traces detected using circular statistics (i) and the subsequent scan analysis (ii) in the absence (*A–C*) and presence (*D–F*) of ventricular contraction. The Rayleigh circular stats analysis tests the distribution of the CaT delay values (blue crosses) along the regular RR interval, presented as a circular event. Traces with *P*_R_ < 0.05 are considered entrained (*Ai* and *Di*), whereas traces with *P*_R_ > 0.05 are considered not-entrained (*Bi*, *Ci*, *Ei*, and *Fi*). (ii) The scan analysis then finds the entrained sections within the trace by finding CaTs where the SD of the Δdelay lays within 2 × SD range. Red circle, entrained CaT; black cross, not-entrained CaT; grey plus; QRS event. For each panel, subpanels (i) and (ii) represent the Rayleigh analysis and scan analysis from the same dataset, respectively.

To find entrainment, the Rayleigh circular statistics test was applied to traces recorded in the absence of electrical pacing. However, only recordings with at least 20 QRS and 5 CaT events were analysed, which was the case for 75% (*n* = 69/92). These traces were analysed using the Rayleigh circular statistics test, of which 30 traces belonged to the contraction recovery group and 39 traces to the no contraction group. From the 69 traces tested, 17 resulted in a *P*_R_ value <0.05 and, on this basis, showed a non-uniform distribution of delay values. Of these, 6 traces were recorded in the absence of detectable ventricular contraction (15%, *n* = 6/39), while 11 traces (37%, *n* = 11/30) were recorded in the presence of some degree of ventricular contraction. *Figure 4* displays examples of the analysis used to find entrainment (Ai and Di) or no entrainment (Bi, Ci, Ei, and Fi). Data of all traces showing a significant test value (*P*_R_ < 0.05) and scan test are shown in *Table 1*.

**Table 1:** Overview of recorded spontaneous traces in the presence and absence of contraction

	Meta data				Experimental data		Circular statistics			Scan test				
Group	Animal number	Time since implantation (min)	*N* QRS events	N CaTs	Median R-R interval (ms)	Contraction (mmHg)	Rayleigh test *P*-value (*P*_R_)	Mean delay (ms)	Mode delay (ms)	*N* entrained	QRS:CaT ratio (*n*:1)	Entrained mean delay (ms)	Entrained SD delay (ms)	Entrained Δdelay (ms)
Contr. recovery	1	71	45	15	362	58.2	0.000	113	136	5	2.25	85	7	5
Contr. recovery	1	95	48	15	363	51	0.000	66	40	6	3	45	12	12
Contr. recovery	2	69	48	27	429	51.2	0.001	85	76	6	1.6	73	8	6
Contr. recovery	3	52	70	17	366	35	0.000	76	65	9	4	63	9	9
Contr. recovery	4	46	81	27	367	17.4	0.000	140	159	10	3	154	9	8
Contr. recovery	6	47	70	35	561	7.3	0.000	111	112	8	2	132	13	12
Contr. recovery	6	47	45	22	562	7.3	0.001	77	42	7	2	39	10	15
Contr. recovery^a^	1	0	30	8	336	0.7	0.005	99	100	Did not find five consecutive CaT within 2 × SD				
Contr. recovery	3	52	40	10	370	35	0.001	224	220	–	–	–	–	–
Contr. recovery	3	98	46	11	344	59	0.000	135	147	–	–	–	–	–
Contr. recovery	5	24	32	15	453	5.2	0.027	356	356	–	–	–	–`	–
No contr.	8	27	36	9	400	0.6	0.001	162	145	5	4	145	6	9
No contr.	8	27	45	11	399	0.6	0.000	12	9	6	4	14	10	10
No contr.	8	50	70	17	416	0.6	0.004	228	165	5	4	165	7	9
No contr.	8	96	69	23	443	0.6	0.000	143	95	8	3	105	14	13
No contr.	8	96	69	23	441	0.6	0.000	48	44	14	3	48	10	9
No contr.	5	22	39	5	358	0.6	0.005	92	102	Did not find five consecutive CaT within 2 × SD				

For all traces in the table, the Rayleigh circular statistical test resulted in a *P* < 0.05 and thus showed a non-uniform distribution and thus potential entrainment (contraction recovery: *n* = 11, *N* = 6; no contraction: *n* = 6, *N* = 2). Only a subset of traces contained five consecutive entrained CaT within their average delay ± 2 × SD (contraction recovery: *n* = 7, *N* = 5; no contraction: *n* = 5, *N* = 1) representing a persistent period of entrainment.

^a^Data obtained from the trace shown in *Figure 3*.

The Rayleigh test indicates the probability of a uniform phase relationship between the pECG and the CaT timing occurring by chance, but the test does not indicate which segment(s) of the trace is entrained. Therefore, the traces showing a significant *P*_R_ were further analysed; each trace was scanned for segments with at least five consecutive CaT events where the difference in consecutive delays (Δdelay) was within a 2 × SD of the Δdelay values. *Figure 4* displays an example analysis of traces that show entrainment (*Aii* and *Dii*) or no entrainment (*Bii*, *Cii*, *Eii*, and *Fii*).

Using this definition, 12 traces from 6 hearts showed periodic entrainment (17%, *n* = 12/69), of which 5 (42%) were from the no contraction group but were all from the same experiment (*N* = 1/9). The other seven (58%) were recorded in the presence of a significant contraction amplitude (*N* = 5/6; *P* = 0.011 vs. no contraction). In cases where there is no entrainment, both the CaT events and the QRS events appear at a regular pace, but independently from each other, resulting in a drift pattern in the value of the delay, albeit slow (*Figure 4Bii* and *Eii*) or fast (*Figure 4Cii* and *Fii*). Notably, traces that show intermittent coupling also show a drift pattern initially, but this is interrupted once the EHT entrains with the pECG and starts beating in a fixed relationship with the pECG for several beats. This is followed by a return to a drifting pattern of delay values when the coupling discontinues (*Figure 4Aii* and *Dii*). Taken together, periodic entrainment is seen during the acute implantation phase, and this is seen more often, but not solely, in the presence of ventricular contraction.

### 3.5 Entrained trace sections are distinct from non-entrained sections

**Figure 5:**
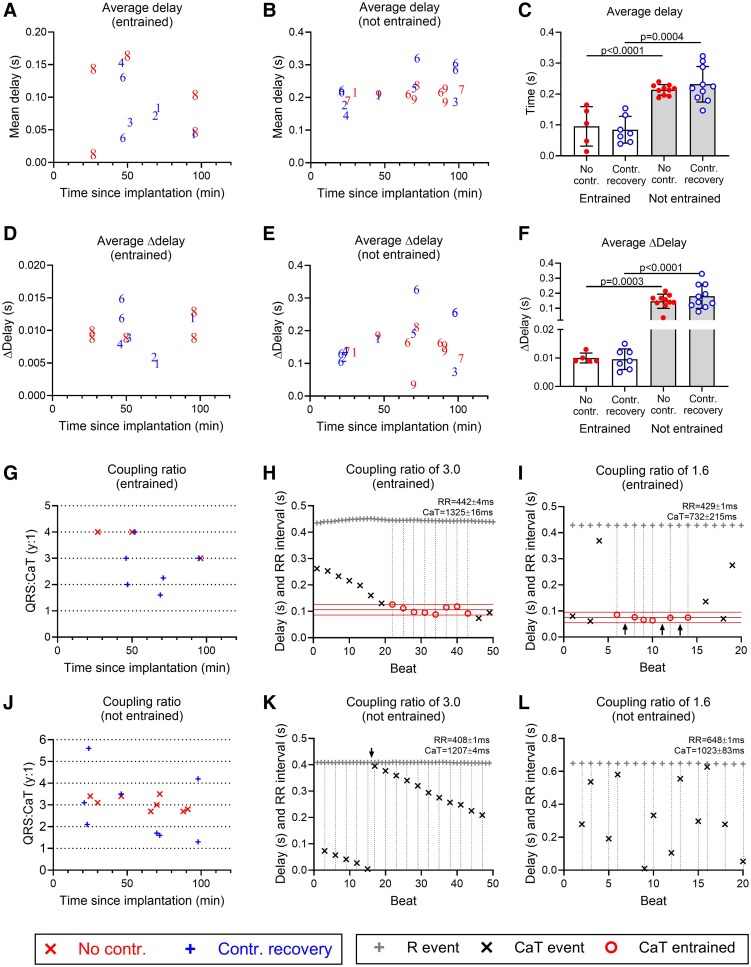
Characterization of entrained and not-entrained trace selections. (*A* and *B*) Average delay values plotted against the implantation time for entrained traces (*A*) and not-entrained traces (*B*). Numbers indicate the animal number for groups in presence (blue) and in absence (red) of ventricular contraction. (*C*) Average delay values. One-way analysis of variance (ANOVA) with Dunnett’s *post hoc* test. Entrained, no contraction: *N* = 1 heart, *n* = 5 traces; entrained, contraction recovery: *N* = 5 hearts, *n* = 7 traces; not-entrained, no contraction: *N* = 5 hearts, *n* = 10 traces; not-entrained, contraction recovery: *N* = 6 hearts; *n* = 10 traces. Entrained vs. not-entrained: no contraction *P* < 0.0001; contraction recovery *P* = 0.0004. (*D* and *E*) Average delta delay (Δdelay) values plotted against implantation time for entrained traces (*D*) and not-entrained traces (*E*). (*F*) Average Δdelay values. One-way ANOVA with Dunnett’s *post hoc* test. Entrained, no contraction: *N* = 1 heart, *n* = 5 traces; entrained, contraction recovery: *N* = 5 hearts, *n* = 7 traces; not-entrained, no contraction: *N* = 5 hearts, *n* = 10 traces; not-entrained, contraction recovery: *N* = 6 hearts; *n* = 10 traces. Entrained vs. not-entrained: no contraction *P* = 0.0003; contraction recovery *P* < 0.0001. (*G*) QRS:CaT coupling ratio for entrained traces in the presence (red) or absence (blue) of LV contraction. (*H* and *I*) Example traces that are entrained. Black arrows indicate missing CaT events. (*J*) QRS:CaT coupling ratio for not-entrained traces in the presence (red) or absence (blue) of LV contraction. (*K* and *L*) Example traces that are not-entrained. Black arrow (*K*) indicates an abrupt change in the delay value due to the drifting pattern of CaT events.

To further assess the characteristics of entrainment, the 12 trace selections showing periodic entrainment were analysed for the average delay value, the average change in delay (Δdelay) and the QRS:CaT coupling ratio. These values were then compared with traces without entrainment, i.e. with the highest *P*_R_ value (10 traces for each experimental group). The results are shown in *Figure 5*.

The mean delay values of entrained trace selections were not significantly different between both groups (no contraction: 95.4 ± 63.8 ms; contraction recovery: 84.4 ± 43.4 ms; *P* = 0.7287; *Figure 5A* and *C*). The combined average of the delay values for all entrained traces was 89 ± 50 ms (*n* = 12). Importantly, the average delay of non-entrained traces was significantly greater than the entrained trace selections (no contraction: 213.5 ± 16.9 ms, *P* < 0.0001; contraction recovery: 231.7 ± 57.4 ms, *P* = 0.0004), as shown in *Figure 5B* and *C*.

*Figure 5D* and *E* shows the average Δdelay for every entrained or non-entrained trace. Plotting all data together in *Figure 5F*, it is clear that the average Δdelay is more than an order of magnitude smaller for entrained trace selections (no contraction: 10.0 ± 1.7 ms; contraction recovery: 9.6 ± 3.6 ms; *P* > 0.99) compared with the not-entrained traces (no contraction: 146.1 ± 47.3 ms; contraction recovery: 179.6 ± 80.2 ms: *P* = 0.50). The difference between entrained and non-entrained Δdelay values was highly significantly different (no contraction: *P* = 0.0003; contraction recovery: *P* < 0.0001). The combined average of the Δdelay values for all entrained traces was 10 ± 3 ms (*n* = 12). These data indicate that in segments when the QRS and CaT signals appear entrained, the difference between consecutive delay values is ∼10 ms.

When trace selections are entrained, the CaT and QRS events should show an exact coupling ratio. Indeed, *Figure 5G* shows that almost all trace selections displayed absolute QRS:CaT ratios between 2:1 and 4:1. An example trace with a coupling ratio of 3:1 is plotted in *Figure 5H* and shows a regular CaT interval with a consistent delay value of ∼100 ms. However, in two traces, the average value coupling ratio was 1.6:1 and 2.2:1. This can be explained by considering the individual CaT events as shown in *Figure 5I*. Despite the regular delay value of again ∼100 ms and the regular RR interval of 420 ms, the coupling ratio varied beat to beat and was sometimes 1:1 and sometimes 2:1, hence the average of 1.6:1. In contrast and as expected, none of the individual coupling ratios of not entrained traces was exact, as all fell in between exact numbers as shown in *Figure 5J*, apart from one trace that resulted in a coupling ratio of 3:1. This specific trace is plotted in *Figure 5K*, and shows that the CaT interval was very regular at a pace close to 3:1, but lacked a consistent delay value. Additionally, a non-entrained trace with a coupling ratio of 1.6:1 is plotted in *Figure 5L* and shows a fast drift pattern that was distinct from the entrained trace plotted in *Figure 5I*. In summary, using this method, we found that the entrained parts of the recorded traces showed consistent features such as a short delay and Δdelay as well as an exact coupling ratio. These were distinct from non-entrained traces.

### 3.6 Intermittent entrainment is unlikely to be caused by GJ formation

One potential cause for intermittent entrainment is GJ formation between the rabbit myocardium and the implanted hiPSC-CMs. If there were to be GJs between these cell types, the Cal520 dye could diffuse from the graft to the host myocardium.^38^ This hypothesis was tested by examining the myocardium for Cal520 signals at the peak of myocardial systole. There was no detectable Cal520 fluorescence in the heart across any of the groups, indicating an absence of functional GJs between the graft and host myocardium. Details of the analysis are found in Supplementary material online, *Methods*, and the results are shown in Supplementary material online, *S6*. In brief, no increase in calcium signal was observed during systole, indicating an absence of functional GJs between the graft and the host myocardium. Therefore, it is unlikely that GJ formation and subsequent isoelectric coupling is the underlying cause of the intermittent entrainment seen during our experiments.

### 3.7 Computational modelling shows field effects might explain the entrainment seen

**Figure 6:**
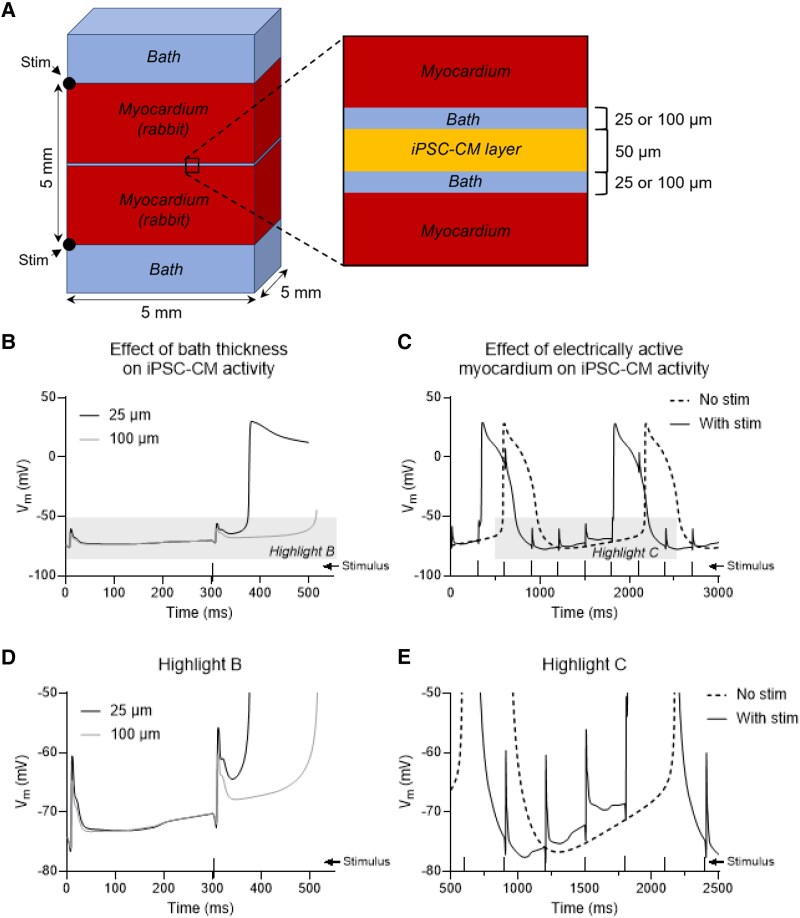
Mathematical model to explore field-effect coupling as potential mechanism for entrainment. (*A*) Schematic 3D diagram of the *in silico* model used, consisting of two myocardial layers [myocardium (rabbit); total 5 × 5 × 5 mm] that are surrounded with a fluid (bath). The myocardium is electrically stimulated from two outer points (black dots). Insert: 2D representation of a subsection of the hiPSC-CM layer (50 μm thick), situated in between the myocardial layers. Bath represents the layer of fluid in between the myocardium and the hiPSC-CM layer and is 25 μm thick under standard conditions but is 100 μm in specified conditions. (*B*) Membrane potential (*V*_m_) of the hiPSC-CM layer with a bath thickness of either 25 μm (black) or 100 μm (grey). Light grey box highlights part of the trace enlarged in (*D*). (*C*) *V*_m_ of the hiPSC-CM layer with a 25 μm bath thickness in the presence (grey) and absence (black) of electrically active myocardium (see Supplementary material online, *Figure S2*). Light grey box highlights part of the trace enlarged in (*E*).

To understand the circumstances that may cause electrical entrainment, a computational model representing the EHT and myocardial implantation site was developed to approximate the electrophysical interactions between a thin (25 μm) layer of hiPSC-CMs and the surrounding bulk myocardium (*Figure 6A* and Supplementary material online, *S3*). *Figure 6B* shows plots of the resulting transmembrane potential (*V*_m_) of a hiPSC-CM tissue (EHT) between two sections of normal rabbit myocardium. Two scenarios were modelled: (i) when the EHT is surrounded by a 25 μm thick layer of saline (grey) or (ii) when the thickness of the layer is 100 μm (black). As shown, when the layer of saline is 25 μm, entrainment from the surrounding myocardium occurred. However, when the bath/saline layer is increased in thickness to 100 μm and entrainment is absent, the lower electric field strength across the saline layer was insufficient to trigger entrainment. To verify that the depolarization of the hiPSC-CM tissue was linked to the electrical activity of the surrounding myocardium, additional simulations were repeated when the surrounding myocardium was not stimulated (*Figure 6C*). Without stimulus, the hiPSC-CM layer does exhibit spontaneous depolarization (grey trace), instead depolarizing every ∼1500 ms, as it would do in the absence of the surrounding myocardium. Entrainment occurred consistently following a paced stimulus to the myocardium, albeit not on every event but in a 4:1 ratio. Note also that the delay between the myocardial electrical event and the firing of an evoked AP in the EHT was of the order of 90–100 ms. As shown in *Figure 6C*, the induced electric field across the tissue cleft causes a limited depolarization that is insufficient to immediately activate an AP in the hiPSC-CMs of the EHT. Instead, it required a second stimulus 300 ms later to cause a further depolarization and activate inward sodium current and rapid depolarization in the EHT. This long coupling delay is also a feature of the entrained records derived from the *ex vivo* measurements reported in this study (89 ± 50 ms), and the cumulative effect of sequential individual field effect influences highlighted in *Figure 6D* and *E* is a feature of field stimulation reported in previous simulations of this phenomenon.^39^

## 4. Discussion

The aim of this study was to investigate the behaviour of mini-EHTs made from SC-CMs during the acute phase (1–2 h) of implantation into the LV of an isolated Langendorff-perfused rabbit heart contracting at sinus rates of 2.0–3.5 Hz. The EHTs displayed lower rates of spontaneous electrical activity (∼0.3 Hz) than the surrounding myocardium and generated fluorescent calcium signals for at least 90 min after implantation, confirming their viability. During this acute phase, the regular pattern of activity of some EHTs was interrupted by short phases of entrainment with the host myocardium with coupling ratios (myocardium:EHT) of 2:1, 3:1, and 4:1. Although entrainment was observed in the absence of mechanical activity, the phenomenon was observed more frequently in contracting ventricles. Computational modelling suggests that myocardial depolarization could initiate depolarization of the EHT through field-effect coupling with similar timing and variability to the experimental data. The mathematical model showed that coupling efficiency is highly dependent on the thickness of the saline film between the two types of tissue. This latter feature may explain the higher incidence of coupling in contracting myocardium, where the film thickness between myocardium and EHT may be minimized by the physical contraction.

### 4.1 Limits to the frequency of stimulation of the isolated small EHTs

Prior to implantation, all isolated EHTs followed external pacing frequencies up to 2.5 Hz but about half could follow 3.3 Hz. This frequency range spans the sinus rates observed in isolated rabbit hearts and indicated that in the event of efficient coupling, the EHT could follow 1:1 but this is at the limit of what the EHT could support. There was a distinct type of response at 3.3 Hz (300 ms cycle length): EHTs either showed alternans of APD or responded to every other stimulus (2:1 block). As pacing frequencies were increased from the spontaneous rate (∼0.3 Hz) to 2.5 Hz, hiPSC-CMs APD90 shortened, demonstrating a similar APD/frequency relationship to that shown experimentally or by *in silico* models in adult human ventricular cells and myocardium.^40–42^ This frequency-dependent APD restitution is a consequence of the kinetics of activation and inactivation processes of Ca^2+^ and Na^+^ currents.^40^ Adult myocardium paced at short cycle lengths (300 ms, 3.3 Hz) commonly shows APD alternans associated with a DI ∼<50–70 ms.^40,43^ This is comparable with the threshold DI for alternans and 2:1 block observed during the EHT pacing protocol (*Figure 2B* and *F*). As shown previously, on increasing pacing frequency, alternans of APD transitioned into a 2:1 block because initially DI shortens to the point that AP repolarization impinges on the cycle length, leading to poor recovery of the Na^+^ current from inactivation and cell/tissue refractoriness.^40^ In comparison, rabbit ventricular APD measurements at 2.5–3.3 Hz indicated that the DI is considerably longer (140–170 ms) due to the shorter APD in the rabbit at these pacing rates, hence the absence of refractory-related phenomena. Previous studies have described the relationship between DI and APD90 in rabbit myocardium and noted that at DI intervals <70 ms achieved at stimulation frequencies of ∼6–7 Hz, 2:1 coupling, and failure to capture become evident. At these higher pacing rates, the recorded DI values are not dissimilar to the minimum DIs tolerated by EHTs but at a lower stimulus rate (3.3 Hz). Thus, based on these data, if there was rapid (<10 ms) and efficient electrical coupling of the rabbit ventricle to engrafted EHT, a 1:1 coupling ratio would be possible at sinus rates of up to ∼3 Hz and coupling ratios of 2:1 would be required for higher sinus rates including 3.3 Hz. Therefore, the rabbit is suitable as a small animal model for studying electrical graft–host coupling. Please note that the inability of the EHT to follow stimulations rates >2.5 Hz (150 b.p.m.) is no limitation to *in vivo* applications since these rates in the human would be associated with significant sympathetic activation and the associated APD shortening would allow higher frequencies to be achieved with stable electrophysiology.

### 4.2 Detection and quantification of episodes of entrainment

Based on the simultaneous recording of ECG signals and EHT-based CaTs to mark the timing of the ventricular and EHT activation, respectively, the Rayleigh test^30,31,44^ detected significant clusters of uniform coupling intervals in a subset of records, suggesting a clear relationship between the myocardial and EHT electrical activity in both contracting and non-contracting myocardium. Subsequent analysis of the timing events within these records revealed segments that satisfied criteria for short periods of entrainment, namely, a minimum of five sequential serial EHT events with coupling delay values (Δdelay) that varied by <2 × SD. On examining the relative entrainment of EHT to ventricular muscle, 1:1 coupling was never observed, but 2:1, 3:1, or 4:1 coupling was present. The analysis further revealed that the sections of sustained entrained activations had a delay between ventricular and EHT activation of up to 165 ms with a mean value of ∼90 ms. The long and variable activation delays may be one of the reasons for the absence of sustained periods of 1:1 coupling, even in hearts with sinus rates as low as 2.5 Hz (400 ms cycle length); the addition of a coupling delay of ∼100 ms to an APD of ∼300 ms leaves no allowance for DI. The 2:1 and 3:1 coupling intervals equate to DIs >400 ms allowing recovery of the excitability of the EHT before the next coupling event. Exposing hiPSC-CMs to a treatment of triiodothyronine (T3) and dexamethasone has been shown to shorten APD^45^ and could therefore improve the coupling ratios during future studies. There may be other coupling phenomena that are not detected through this analysis. For example, in at least one preparation, Wenckebach-like phenomenon of progressively increasing coupling delay was observed, but the Rayleigh test failed to show significance. Methods that would allow longer periods of monitoring would be required for more complex forms of coupling such as these to be verified.

### 4.3 Intermittent coupling in the presence and absence of ventricular contraction

Records of sustained entrainment were observed in 5/6 (83%) of experiments using EHTs in contracting myocardium. However, the generation of significant pressures within the LV lumen and myocardium through ventricular contraction was not an absolute requirement for entrainment since in 1/9 (11%) experiments showed several instances of sustained coupling, and one other engrafted heart showed a significant cluster of coupling intervals but did not satisfy the criteria for a sustained train (*Table 1*). The delay and variability of the coupling observed in the two situations (contracting/non-contracting) were not significantly different, indicating that a similar coupling process was involved but with much lower probability in mechanically quiescent preparations. More details on the *in situ* physical arrangement of EHT and myocardium would be required to gain further insight into the factors that favour electrical coupling. Moreover, improvement of imaging technologies that allowed longer term monitoring of EHT activity to improve the assessment of the host-EHT coupling both *ex vivo* and potentially *in vivo*.

### 4.4 Field-effect phenomena rather than GJ coupling or mechano-electrical feedback as a potential mechanism for entrainment

GJs are formed by pairs of connexins (Cx) hemichannels and are responsible for AP propagation in adult myocardium. Both the rabbit adult cardiomyocytes and the implanted immature hiPSC-CMs express Cx43, but the expression levels and Cx43 distribution are different in both cell types.^46^ In adult myocytes, the turnover of Cx43 molecules is ∼2 h,^47,48^ similar to our experimental timeline. Moreover, in certain *in vitro* conditions, hiPSC-CMs were able to establish conduction within 2 h^24^ and adult CMs in an even shorter timeframe.^22,23^ So, in theory, GJs could be the underlying mechanism for entrainment. Performing immunohistochemical staining for Cx43 can localized GJs, but this does not indicate functionality. Moreover, only the resolution of electron microscopy can confirm channel formation.^22,23^ Instead, we chose to test for the functionality of GJ coupling by assessing the diffusion of small molecules from the EHT to the myocardium.^38^ Previous studies of dye transfer through GJs have suggested the half-time for diffusion in myocardium is 5–10 min.^49^ On this basis, the transfer of Cal520 (similar molecular size to the more commonly used Calcein) from EHT to myocardium should be detectable as the emergence of a CaT in synchrony with the pECG and a loss of the signal from the EHT. As shown in Supplementary material online, *S6*, careful examination of the fluorescence signals from the implantation site failed to show CaT complexes synchronized to the pECG complex. This suggests there were no functional GJs between EHT and myocardium to support diffusion. Therefore, it is unlikely that GJ formation between EHT and myocardium would be the basis for the intermittent coupling patterns seen in this study.

Mechano-electrical feedback (MEF) is a well-studied phenomenon in adult myocardium^50^ describing the influence of the mechanical state of the myocardium on the electrical activity, some of which is mediated by stretch-activated channels. This physiological mechanism is a potential link between contraction of the host myocardium and the electrical activation of the EHT. This hypothesis was directly tested in this study using a contraction inhibitor (blebbistatin) which reduced to <2% the mechanical activity of the heart without substantially influencing the electrophysiology of the myocardium at sinus rate.^51^ Under these conditions, entrainment was observed in 2/9 and sustained entrainment in 1/9 preparations. These preparations showed entrainment characteristics that were not quantitatively different from that seen more regularly when contraction was present in terms of the magnitude of the delay or the consistency of the delay. This observation suggests that mechanical activity is not a requirement of—but does enhance the coupling mechanism between—EHT and myocardium observed in this study, indicating the MEF is not the sole cause. This logic would indicate that stretch-activated channels or other mechano-electric feedback mechanisms are not key to this entrainment mechanism.

Another mechanism that appears feasible from the literature is the existence of non-myocyte bridges between the EHT and the host myocardium, e.g. cardiac fibroblasts.^52,53^ These cells use GJ mechanisms to establish electrical coupling across distances of up to 0.3 mm.^54^ As with the other cellular mechanisms, the short timescales of this study would make these explanations unlikely.

Another potential mechanism that could explain the observed entrainment is electric field-mediated coupling, whereby the electrical activity of the neighbouring myocardium acts as an electrode, causing a localized change in the electric field around the tissue of interest, which in turn causes transmembrane currents to flow and thus depolarizes the membrane of the EHT without involvement of GJs.^39,55^ Cleft width, and therefore gap resistance, is an important determinant of the magnitude of field effects described above.^56^ It should be noted that the field-effect process described above is distinct but related to the process of ephaptic coupling evoked to explain electrical coupling across the perinexus between two physically coupled cardiomyocytes, a distinction made previously within neurophysiology.^57^

In our study, the biophysical situation of the grafted cells surrounded by the myocardium was examined with a computational model using the specific properties of both cell types (adult rabbit ventricle CMs vs. hiPSC-CMs). Using these parameters, the model demonstrated that the field-effect phenomenon has the capability to entrain the electrical activity of the EHT. The modelling reproduced several aspects of the coupling observed experimentally: (i) the coupling delay was variable and could be up to 100–150 ms, (ii) the coupling mechanism required a long DI, (iii) the efficiency of the field-effect coupling was critically dependent on the thickness of the fluid film between the two tissues, and (iv) the spontaneous depolarization of hiPSC-CMs during diastole. While this latter aspect was not explicitly measured in this study, this critical variable may explain the relatively unreliable nature of the coupling observed, including the higher incidence of entrainment in contracting myocardium where the thickness of the saline film between myocardium and EHT may be minimized by the higher pressure within the contracting ventricle wall and therefore the forces tending to minimize the gap within the cleft sandwiching the EHT.

Earlier *in silico* studies modelling hiPSC-CM clusters within a myocardial scar have indicated that field-effect phenomena are a potential coupling mechanism.^56^ However, this is the first report to provide experimental evidence of field-effect coupling linking EHTs to host myocardium. Furthermore, a recent experimental study showed evidence of the ability of cardiac fibroblasts lacking GJs to electrically couple regions of adult CMs,^58^ and again parallel *in silico* modelling showed that field-effect phenomena could be the underlying mechanism.^58^ The current study emphasizes the relatively large intrinsic delay and consequent inefficiencies of this form of coupling compared with what would be achieved through the low-resistance GJ-mediated coupling, which would be required for long-term high-fidelity electrical coupling of the EHT to the myocardium.

### 4.5 Absence of ventricular arrhythmias

It is interesting to note that during this initial phase, the entrainment of the EHTs was not associated with any additional arrhythmias or ectopic beats, indicating that the presence of the EHT itself is not proarrhythmic. Xie *et al.*^59^ estimated 10 times the number of cells used in this study to be required to initiate an ectopic (600k). Experimental evidence has shown that 56 days after implantation 30 million hiPSC-CMs can indeed activate the myocardium, a number almost 1000× larger.^60^ So, the absence of EHT-linked ectopic beats, in our study, is consistent with the small cell number within the EHT and supports the hypothesis that the hiPSC-CM entrainment was due to field effects generated by the much larger electrical mass of the myocardium. This principle could not apply in reverse unless the graft size was considerably larger^59,60^ and the adult myocardium displayed the same Phase 4 spontaneous depolarization as the hiPSC-CM. The same principle underlies the process of ephaptic coupling, a form of electrical coupling or transmission that is thought to operate between adult cardiac myocytes across the intercalated disc under certain circumstances.^55,61,62^ In this latter form of coupling, however, the gap between two cells is 10–20 nm, considerably less than the ∼20 µm thought to occur between the EHT and host myocardium in our study.

**Translational perspective**

Cardiac regeneration therapies have the potential to re-muscularize a myocardial scar. One of the challenges in the field is understanding the processes required to establish electrical coupling between host myocardium and graft tissue, a key step to successful mechanical and electrical integration. This experimental study shows that grafts of cultured heart cells can entrain intermittently with the host ventricle <1 h post implant. The entrainment appears to be mediated by transient electrical fields generated by the surrounding host myocardium that influences the electrical activity of the graft. This early electrical entrainment may be important in establishing the long-term integration of the graft with the myocardium, thereby maximize the benefit of regenerative therapy.

## 5. Conclusion

This work examined the behaviour of EHTs grafted sub-epicardially in rabbit LV to study the spontaneous activity and potential coupling to the myocardium over the initial 90–120 min. Brief periods of electrical entrainment were observed, which have the characteristics of field-effect-mediated stimulation of the EHT by the host myocardium. Further studies are required to distinguish what role this form of coupling may have in the mechanical and electrical integration of the EHT over longer periods and the extent to which it may prevent or initiate arrhythmias early after EHT implantation. This work builds up our understanding of the host–graft interactions that occur in normal myocardium shortly after implantation and will allow us to compare with equivalent grafting in post-MI models. This early electrical entrainment may be important in establishing the long-term integration of the graft with the myocardium, thereby maximizing the benefit of regenerative therapy.

## Supplementary Material

cvaf034_Supplementary_Data

## Data Availability

The data underlying this research will be shared on reasonable request with the corresponding author.

## References

[1] Breckwoldt K, Letuffe-Brenière D, Mannhardt I, Schulze T, Ulmer B, Werner T, Benzin A, Klampe B, Reinsch MC, Laufer S, Shibamiya A, Prondzynski M, Mearini G, Schade D, Fuchs S, Neuber C, Krämer E, Saleem U, Schulze ML, Rodriguez ML, Eschenhagen T, Hansen A. Differentiation of cardiomyocytes and generation of human engineered heart tissue. Nat Protoc 2017;12:1177–1197. 28492526 10.1038/nprot.2017.033

[2] Takahashi K, Tanabe K, Ohnuki M, Narita M, Ichisaka T, Tomoda K, Yamanaka S. Induction of pluripotent stem cells from adult human fibroblasts by defined factors. Cell 2007;131:861–872. 18035408 10.1016/j.cell.2007.11.019

[3] Tohyama S, Hattori F, Sano M, Hishiki T, Nagahata Y, Matsuura T, Hashimoto H, Suzuki T, Yamashita H, Satoh Y, Egashira T, Seki T, Muraoka N, Yamakawa H, Ohgino Y, Tanaka T, Yoichi M, Yuasa S, Murata M, Suematsu M, Fukuda K. Distinct metabolic flow enables large-scale purification of mouse and human pluripotent stem cell-derived cardiomyocytes. Cell Stem Cell 2013;12:127–137. 23168164 10.1016/j.stem.2012.09.013

[4] Weinberger F, Eschenhagen T. Cardiac regeneration: new hope for an old dream. Annu Rev Physiol 2021;83:59–81. 33064963 10.1146/annurev-physiol-031120-103629

[5] Miyagawa S, Kainuma S, Kawamura T, Suzuki K, Ito Y, Iseoka H, Ito E, Takeda M, Sasai M, Mochizuki-Oda N, Shimamoto T, Nitta Y, Dohi H, Watabe T, Sakata Y, Toda K, Sawa Y. Case report: transplantation of human induced pluripotent stem cell-derived cardiomyocyte patches for ischemic cardiomyopathy. Front Cardiovasc Med 2022;9. 10.3389/fcvm.2022.950829 PMC9426776 36051285

[6] Shiba Y, Fernandes S, Zhu W-Z, Filice D, Muskheli V, Kim J, Palpant NJ, Gantz J, Moyes KW, Reinecke H, Van Biber B, Dardas T, Mignone JL, Izawa A, Hanna R, Viswanathan M, Gold JD, Kotlikoff MI, Sarvazyan N, Kay MW, Murry CE, Laflamme MA. Human ES-cell-derived cardiomyocytes electrically couple and suppress arrhythmias in injured hearts. Nature 2012;489:322–325. 22864415 10.1038/nature11317 PMC3443324

[7] Chong JJH, Yang X, Don CW, Minami E, Liu Y-W, Weyers JJ, Mahoney WM, Van Biber B, Cook SM, Palpant NJ, Gantz JA, Fugate JA, Muskheli V, Gough GM, Vogel KW, Astley CA, Hotchkiss CE, Baldessari A, Pabon L, Reinecke H, Gill EA, Nelson V, Kiem H-P, Laflamme MA, Murry CE. Human embryonic-stem-cell-derived cardiomyocytes regenerate non-human primate hearts. Nature 2014;510:273–277. 24776797 10.1038/nature13233 PMC4154594

[8] Gerbin KA, Yang X, Murry CE, Coulombe KLK. Enhanced electrical integration of engineered human myocardium via intramyocardial versus epicardial delivery in infarcted rat hearts. PLoS One 2015;10:e0131446. 26161513 10.1371/journal.pone.0131446 PMC4498815

[9] Shiba Y, Gomibuchi T, Seto T, Wada Y, Ichimura H, Tanaka Y, Ogasawara T, Okada K, Shiba N, Sakamoto K, Ido D, Shiina T, Ohkura M, Nakai J, Uno N, Kazuki Y, Oshimura M, Minami I, Ikeda U. Allogeneic transplantation of iPS cell-derived cardiomyocytes regenerates primate hearts. Nature 2016;538:388–391. 27723741 10.1038/nature19815

[10] Rubart M, Pasumarthi KBS, Nakajima H, Soonpaa MH, Nakajima HO, Field LJ. Physiological coupling of donor and host cardiomyocytes after cellular transplantation. Circ Res 2003;92:1217–1224. 12730096 10.1161/01.RES.0000075089.39335.8C

[11] Jackman CP, Ganapathi AM, Asfour H, Qian Y, Allen BW, Li Y, Bursac N. Engineered cardiac tissue patch maintains structural and electrical properties after epicardial implantation. Biomaterials 2018;159:48–58. 29309993 10.1016/j.biomaterials.2018.01.002 PMC5801076

[12] Weinberger F, Breckwoldt K, Pecha S, Kelly A, Geertz B, Starbatty J, Yorgan T, Cheng K-H, Lessmann K, Stolen T, Scherrer-Crosbie M, Smith G, Reichenspurner H, Hansen A, Eschenhagen T. Cardiac repair in guinea pigs with human engineered heart tissue from induced pluripotent stem cells. Sci Transl Med 2016;8:363ra148. 10.1126/scitranslmed.aaf8781 27807283

[13] Jabbour RJ, Owen TJ, Pandey P, Reinsch M, Wang B, King O, Couch LS, Pantou D, Pitcher DS, Chowdhury RA, Pitoulis FG, Handa BS, Kit-Anan W, Perbellini F, Myles RC, Stuckey DJ, Dunne M, Shanmuganathan M, Peters NS, Ng FS, Weinberger F, Terracciano CM, Smith GL, Eschenhagen T, Harding SE. In vivo grafting of large engineered heart tissue patches for cardiac repair. JCI Insight 2021;6:e144068. 34369384 10.1172/jci.insight.144068 PMC8410032

[14] Guragain B, Wei Y, Zhang H, Kahn-Krell A, Ye L, Walcott GP, Rogers JM, Zhang JJ. Implanted human cardiac spheroids electrically couple with infarcted swine myocardium. Circulation 2024;149:1855–1857. 38829934 10.1161/CIRCULATIONAHA.123.068568 PMC11149905

[15] Kobayashi H, Tohyama S, Ichimura H, Ohashi N, Chino S, Soma Y, Tani H, Tanaka Y, Yang X, Shiba N, Kadota S, Haga K, Moriwaki T, Morita-Umei Y, Umei TC, Sekine O, Kishino Y, Kanazawa H, Kawagishi H, Yamada M, Narita K, Naito T, Seto T, Kuwahara K, Shiba Y, Fukuda K. Regeneration of nonhuman primate hearts with human induced pluripotent stem cell-derived cardiac spheroids. Circulation 2024;150:611–621. 38666382 10.1161/CIRCULATIONAHA.123.064876

[16] Romagnuolo R, Masoudpour H, Porta-Sánchez A, Qiang B, Barry J, Laskary A, Qi X, Massé S, Magtibay K, Kawajiri H, Wu J, Valdman Sadikov T, Rothberg J, Panchalingam KM, Titus E, Li R-K, Zandstra PW, Wright GA, Nanthakumar K, Ghugre NR, Keller G, Laflamme MA. Human embryonic stem cell-derived cardiomyocytes regenerate the infarcted pig heart but induce ventricular tachyarrhythmias. Stem Cell Reports 2019;12:967–981. 31056479 10.1016/j.stemcr.2019.04.005 PMC6524945

[17] Liu Y-W, Chen B, Yang X, Fugate JA, Kalucki FA, Futakuchi-Tsuchida A, Couture L, Vogel KW, Astley CA, Baldessari A, Ogle J, Don CW, Steinberg ZL, Seslar SP, Tuck SA, Tsuchida H, Naumova AV, Dupras SK, Lyu MS, Lee J, Hailey DW, Reinecke H, Pabon L, Fryer BH, MacLellan WR, Thies RS, Murry CE. Human embryonic stem cell-derived cardiomyocytes restore function in infarcted hearts of non-human primates. Nat Biotechnol 2018;36:597–605. 29969440 10.1038/nbt.4162 PMC6329375

[18] Gibbs CE, Marchianó S, Zhang K, Yang X, Murry CE, Boyle PM. Graft-host coupling changes can lead to engraftment arrhythmia: a computational study. J Physiol 2023;601:2733–2749. 37014103 10.1113/JP284244 PMC10901678

[19] Kawaguchi S, Soma Y, Nakajima K, Kanazawa H, Tohyama S, Tabei R, Hirano A, Handa N, Yamada Y, Okuda S, Hishikawa S, Teratani T, Kunita S, Kishino Y, Okada M, Tanosaki S, Someya S, Morita Y, Tani H, Kawai Y, Yamazaki M, Ito A, Shibata R, Murohara T, Tabata Y, Kobayashi E, Shimizu H, Fukuda K, Fujita J. Intramyocardial transplantation of human iPS cell-derived cardiac spheroids improves cardiac function in heart failure animals. JACC Basic Transl Sci 2021;6:239–254. 33778211 10.1016/j.jacbts.2020.11.017 PMC7987543

[20] Quinn TA, Kohl P. Cardiac mechano-electric coupling: acute effects of mechanical stimulation on heart rate and rhythm. Physiol Rev 2021;101:37–92. 32380895 10.1152/physrev.00036.2019

[21] Clapham DE, Shrier A, DeHaan RL. Junctional resistance and action potential delay between embryonic heart cell aggregates. J Gen Physiol 1980;75:633–654. 7391810 10.1085/jgp.75.6.633 PMC2215265

[22] Rook MB, Jongsma HJ, van Ginneken AC. Properties of single gap junctional channels between isolated neonatal rat heart cells. Am J Physiol 1988;255:H770–H782. 2459974 10.1152/ajpheart.1988.255.4.H770

[23] Rook MB, de Jonge B, Jongsma HJ, Masson-Pévet MA. Gap junction formation and functional interaction between neonatal rat cardiocytes in culture: a correlative physiological and ultrastructural study. J Membr Biol 1990;118:179–192. 2266548 10.1007/BF01868475

[24] Li J, Minami I, Shiozaki M, Yu L, Yajima S, Miyagawa S, Shiba Y, Morone N, Fukushima S, Yoshioka M, Li S, Qiao J, Li X, Wang L, Kotera H, Nakatsuji N, Sawa Y, Chen Y, Liu L. Human pluripotent stem cell-derived cardiac tissue-like constructs for repairing the infarcted myocardium. Stem Cell Reports 2017;9:1546–1559. 29107590 10.1016/j.stemcr.2017.09.007 PMC5829319

[25] Maxwell MP, Hearse DJ, Yellon DM. Species variation in the coronary collateral circulation during regional myocardial ischaemia: a critical determinant of the rate of evolution and extent of myocardial infarction. Cardiovasc Res 1987;21:737–746. 3440266 10.1093/cvr/21.10.737

[26] Myles RC, Burton FL, Cobbe SM, Smith GL. Alternans of action potential duration and amplitude in rabbits with left ventricular dysfunction following myocardial infarction. J Mol Cell Cardiol 2011;50:510–521. 21145895 10.1016/j.yjmcc.2010.11.019

[27] Morotti S, Liu C, Hegyi B, Ni H, Fogli Iseppe A, Wang L, Pritoni M, Ripplinger CM, Bers DM, Edwards AG, Grandi E. Quantitative cross-species translators of cardiac myocyte electrophysiology: model training, experimental validation, and applications. Sci Adv 2021;7:eabg0927. 34788089 10.1126/sciadv.abg0927 PMC8598003

[28] Huo J, Kamalakar A, Yang X, Word B, Stockbridge N, Lyn-Cook B, Pang L. Evaluation of batch variations in induced pluripotent stem cell-derived human cardiomyocytes from 2 major suppliers. Toxicol Sci 2017;156:25–38. 28031415 10.1093/toxsci/kfw235

[29] Paci M, Sartiani L, Del Lungo M, Jaconi M, Mugelli A, Cerbai E, Severi S. Mathematical modelling of the action potential of human embryonic stem cell derived cardiomyocytes. Biomed Eng Online 2012;11:61. 22929020 10.1186/1475-925X-11-61 PMC3477113

[30] Berens P . CircStat: a MATLAB toolbox for circular statistics. J Stat Softw 2009;31:1–21.

[31] Fisher NI . Statistical Analysis of Circular Data. Cambridge: Cambridge University Press; 1993.

[32] Huethorst E, Mortensen P, Simitev RD, Gao H, Pohjolainen L, Talman V, Ruskoaho H, Burton FL, Gadegaard N, Smith GL. Conventional rigid 2D substrates cause complex contractile signals in monolayers of human induced pluripotent stem cell-derived cardiomyocytes. J Physiol 2022;600:483–507. 34761809 10.1113/JP282228 PMC9299844

[33] Sala L, van Meer BJ, Tertoolen LGJ, Bakkers J, Bellin M, Davis RP, Denning C, Dieben MAE, Eschenhagen T, Giacomelli E, Grandela C, Hansen A, Holman ER, Jongbloed MRM, Kamel SM, Koopman CD, Lachaud Q, Mannhardt I, Mol MPH, Mosqueira D, Orlova VV, Passier R, Ribeiro MC, Saleem U, Smith GL, Burton FL, Mummery CL. MUSCLEMOTION: a versatile open software tool to quantify cardiomyocyte and cardiac muscle contraction in vitro and in vivo. Circ Res 2018;122:e5–e16. 29282212 10.1161/CIRCRESAHA.117.312067 PMC5805275

[34] Robertson T, Cryer JD. An iterative procedure for estimating the mode. J Am Stat Assoc 1974;69:1012–1016.

[35] Mahajan A, Shiferaw Y, Sato D, Baher A, Olcese R, Xie L-H, Yang M-J, Chen P-S, Restrepo JG, Karma A, Garfinkel A, Qu Z, Weiss JN. A rabbit ventricular action potential model replicating cardiac dynamics at rapid heart rates. Biophys J 2008;94:392–410. 18160660 10.1529/biophysj.106.98160 PMC2157228

[36] Forouzandehmehr M, Koivumäki JT, Hyttinen J, Paci M. A mathematical model of hiPSC cardiomyocytes electromechanics. Physiol Rep 2021;9:e15124. 34825519 10.14814/phy2.15124 PMC8617339

[37] Plank G, Loewe A, Neic A, Augustin C, Huang Y-L, Gsell MAF, Karabelas E, Nothstein M, Prassl AJ, Sánchez J, Seemann G, Vigmond EJ. The openCARP simulation environment for cardiac electrophysiology. Comput Methods Programs Biomed 2021;208:106223. 34171774 10.1016/j.cmpb.2021.106223

[38] Adams WP, Raisch TB, Zhao Y, Davalos R, Barrett S, King DR, Bain CB, Colucci-Chang K, Blair GA, Hanlon A, Lozano A, Veeraraghavan R, Wan X, Deschenes I, Smyth JW, Hoeker GS, Gourdie RG, Poelzing S. Extracellular perinexal separation is a principal determinant of cardiac conduction. Circ Res 2023;133:658–673. 37681314 10.1161/CIRCRESAHA.123.322567 PMC10561697

[39] Tung L, Borderies JR. Analysis of electric field stimulation of single cardiac muscle cells. Biophys J 1992;63:371–386. 1420884 10.1016/S0006-3495(92)81632-6 PMC1262161

[40] O'Hara T, Virág L, Varró A, Rudy Y. Simulation of the undiseased human cardiac ventricular action potential: model formulation and experimental validation. PLoS Comput Biol 2011;7:e1002061. 21637795 10.1371/journal.pcbi.1002061 PMC3102752

[41] Hortigon-Vinagre MP, Zamora V, Burton FL, Green J, Gintant GA, Smith GL. The use of ratiometric fluorescence measurements of the voltage sensitive dye di-4-ANEPPS to examine action potential characteristics and drug effects on human induced pluripotent stem cell-derived cardiomyocytes. Toxicol Sci 2016;154:320–331. 27621282 10.1093/toxsci/kfw171 PMC5139069

[42] Franz MR, Swerdlow CD, Liem LB, Schaefer J. Cycle length dependence of human action potential duration in vivo. Effects of single extrastimuli, sudden sustained rate acceleration and deceleration, and different steady-state frequencies. J Clin Invest 1988;82:972–979. 3417875 10.1172/JCI113706 PMC303610

[43] Taggart P, Sutton PMI, Boyett MR, Lab M, Swanton H. Human ventricular action potential duration during short and long cycles. Circulation 1996;94:2526–2534. 8921797 10.1161/01.cir.94.10.2526

[44] Rana KD, Vaina LM, Hämäläinen MS. A fast statistical significance test for baseline correction and comparative analysis in phase locking. Front Neuroinform 2013;7:3. 23919088 10.3389/fninf.2013.00003 PMC3573346

[45] Wang L, Wada Y, Ballan N, Schmeckpeper J, Huang J, Rau CD, Wang Y, Gepstein L, Knollmann BC. Triiodothyronine and dexamethasone alter potassium channel expression and promote electrophysiological maturation of human-induced pluripotent stem cell-derived cardiomyocytes. J Mol Cell Cardiol 2021;161:130–138. 34400182 10.1016/j.yjmcc.2021.08.005 PMC9809541

[46] Karbassi E, Fenix A, Marchiano S, Muraoka N, Nakamura K, Yang X, Murry CE. Cardiomyocyte maturation: advances in knowledge and implications for regenerative medicine. Nat Rev Cardiol 2020;17:341–359. 32015528 10.1038/s41569-019-0331-x PMC7239749

[47] Shaw RM, Fay AJ, Puthenveedu MA, von Zastrow M, Jan Y-N, Jan LY. Microtubule plus-end-tracking proteins target gap junctions directly from the cell interior to adherens junctions. Cell 2007;128:547–560. 17289573 10.1016/j.cell.2006.12.037 PMC1955433

[48] Laird DW, Puranam KL, Revel JP. Turnover and phosphorylation dynamics of connexin43 gap junction protein in cultured cardiac myocytes. Biochem J 1991;273(pt. 1):67–72. 10.1042/bj2730067 PMC1149880 1846532

[49] Lang D, Sato D, Jiang Y, Ginsburg KS, Ripplinger CM, Bers DM. Calcium-dependent arrhythmogenic foci created by weakly coupled myocytes in the failing heart. Circ Res 2017;121:1379–1391. 28970285 10.1161/CIRCRESAHA.117.312050 PMC5722688

[50] Kohl P, Nesbitt AD, Cooper PJ, Lei M. Sudden cardiac death by Commotio cordis: role of mechano-electric feedback. Cardiovasc Res 2001;50:280–289. 11334832 10.1016/s0008-6363(01)00194-8

[51] Fedorov VV, Lozinsky IT, Sosunov EA, Anyukhovsky EP, Rosen MR, Balke CW, Efimov IR. Application of blebbistatin as an excitation–contraction uncoupler for electrophysiologic study of rat and rabbit hearts. Heart Rhythm 2007;4:619–626. 17467631 10.1016/j.hrthm.2006.12.047

[52] Ongstad E, Kohl P. Fibroblast-myocyte coupling in the heart: potential relevance for therapeutic interventions. J Mol Cell Cardiol 2016;91:238–246. 26774702 10.1016/j.yjmcc.2016.01.010 PMC5022561

[53] Rook MB, van Ginneken AC, de Jonge B, el Aoumari A, Gros D, Jongsma HJ. Differences in gap junction channels between cardiac myocytes, fibroblasts, and heterologous pairs. Am J Physiol 1992;263:C959–C977. 1279981 10.1152/ajpcell.1992.263.5.C959

[54] Gaudesius G, Miragoli M, Thomas SP, Rohr S. Coupling of cardiac electrical activity over extended distances by fibroblasts of cardiac origin. Circ Res 2003;93:421–428. 12893743 10.1161/01.RES.0000089258.40661.0C

[55] Lin J, Keener JP. Ephaptic coupling in cardiac myocytes. IEEE Trans Biomed Eng 2013;60:576–582. 23335235 10.1109/TBME.2012.2226720

[56] Yu JK, Liang JA, Weinberg SH, Trayanova NA. Computational modeling of aberrant electrical activity following remuscularization with intramyocardially injected pluripotent stem cell-derived cardiomyocytes. J Mol Cell Cardiol 2022;162:97–109. 34487753 10.1016/j.yjmcc.2021.08.011 PMC8766907

[57] Jefferys JG . Nonsynaptic modulation of neuronal activity in the brain: electric currents and extracellular ions. Physiol Rev 1995;75:689–723. 7480159 10.1152/physrev.1995.75.4.689

[58] Wang Y, Li Q, Tao B, Angelini M, Ramadoss S, Sun B, Wang P, Krokhaleva Y, Ma F, Gu Y, Espinoza A, Yamauchi K, Pellegrini M, Novitch B, Olcese R, Qu Z, Song Z, Deb A. Fibroblasts in heart scar tissue directly regulate cardiac excitability and arrhythmogenesis. Science 2023;381:1480–1487. 37769108 10.1126/science.adh9925 PMC10768850

[59] Xie Y, Sato D, Garfinkel A, Qu Z, Weiss JN. So little source, so much sink: requirements for afterdepolarizations to propagate in tissue. Biophys J 2010;99:1408–1415. 20816052 10.1016/j.bpj.2010.06.042 PMC2931729

[60] Stüdemann T, Schwarzová B, Schneidewind T, Geertz B, von Bibra C, Nehring M, Rössinger J, Wiegert JS, Eschenhagen T, Weinberger F. Impulse initiation in engrafted pluripotent stem cell-derived cardiomyocytes can stimulate the recipient heart. Stem Cell Reports 2024;19:1053–1060. 39059379 10.1016/j.stemcr.2024.06.012 PMC11368679

[61] Veeraraghavan R, Lin J, Keener JP, Gourdie R, Poelzing S. Potassium channels in the Cx43 gap junction perinexus modulate ephaptic coupling: an experimental and modeling study. Pflugers Arch 2016;468:1651–1661. 27510622 10.1007/s00424-016-1861-2 PMC5131566

[62] Lin J, Abraham A, George SA, Greer-Short A, Blair GA, Moreno A, Alber BR, Kay MW, Poelzing S. Ephaptic coupling is a mechanism of conduction reserve during reduced gap junction coupling. Front Physiol 2022;13:848019. 35600295 10.3389/fphys.2022.848019 PMC9117633

