# Supplementary material for: Evidence for intermittent coupling of intramyocardial small, engineered heart tissues acutely implanted into rabbit myocardium
Source: Cardiovasc Res. 2025 Feb 28;121(11):1697–711. doi: 10.1093/cvr/cvaf034 (PMC12477673; doi:10.1093/cvr/cvaf034)
Supplement: cvaf034_Supplementary_Data [file cvaf034_supplementary_data.docx]

## Supplementary methods

### Assessment of Cal520 traces for myocardial interaction

The CaTs from each experimental group were averaged over each CaT-CaT interval (CaT_EHT_; hiPSC-CM) and over each R-R interval of the pECG (CaT_avR_; rabbit CMs). For the latter, only R-R intervals during EHT diastole were selected to assure any changes in fluorescence were caused by myocardial activity. Then, the (F_P_ - F_0_)/F_0_, also described as ∆F/F_0_, was calculated, where the F_0_ represents fluorescent signal during diastole and F_P_ represents the peak fluorescent signal during systole.

For hiPSC-CMs (F), the systolic fluorescent signal was taken at the peak of the CaT_EHT_, defined as F_P(EHT)_. For the CMs (F’), the timepoint of the systolic fluorescent signal (F’_P(heart)_) was first determined using a previous dataset of Langendorff perfused rabbit hearts loaded with Rhod2-AM (N=3 hearts, paced at 2Hz), kindly provided by Dr Cherry Alexander ^1^. Each trace consisted of 8 consecutive CaTs, of which the time from the R-peak of the pECG to the peak of the CaT was averaged. From three hearts, the average delay was 28±2ms (**Supplementary Figure 6A**). So, from CaT_avR_ , peak values were taken 28ms after the R-peak of the pECG.

### Histology

After the Langendorff experiment, part of the LV including the implantation site was removed and submerged in 4% PFA followed by 2 washes with PBS. Samples were then embedded in paraffin and processed for histological staining using a mouse-anti-Ku80 antibody (ab119935, Abcam) and anti-mouse DAB-conjugated secondary antibody. All sections were background stained with haematoxylin.

## Supplementary materials 1

### Cal520 is brighter than Cal590

Initially a dual fluoroscopic imaging approach was tested to be able to record the activity of the EHT (Cal590-AM) and the surrounding myocardium (FluoVolt) simultaneously. Other combinations (e.g. RH237, Fluo-4) were not applicable due to their overlapping ex/em spectrum or because of their low signal to noise ratio. A high signal to noise ratio is crucial to be able to record signals from ~50.000 cells within the EHT through a flap of myocardium that, in some cases, is contracting.

The brightness of Cal590-AM and Cal520-AM were assessed in vitro using the small EHTs of CaT were recorded on the CellOPTIQ system first, followed by the lightguide that is part of the Langendorff perfusion rig. Results are shown in **Supplementary Figure 1A and B**, respectively.

Using high NA objectives on the CellOPTIQ system, it is clear that Cal590 is less bright then Cal520. However, when using the lightguide, Cal590 is near-undetectable and is thus unsuitable for subsequent ex vivo studies. In contrast, Cal520 has shown to be much brighter and therefore more suitable for the implantation studies. So, EHTs will be stained with Cal520-AM before implantation into the heart.


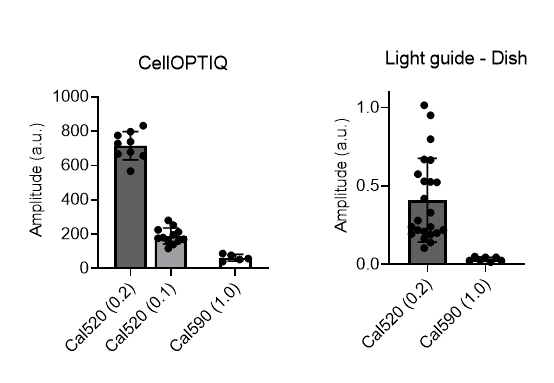


A

B

Figure 1 The difference in brightness between Cal520 and Cal590 calcium indicators when measured in vitro using the CellOPTIQ system (NA=0.6) (A) and using the light guide (NA=0.2) (B). Number behind every dye-indication represents the LED setting. Values of the CellOPTIQ and whole heart set-up cannot be compared 1:1. Error bars represent SD. A) Cal520 (0.2): n=9 EHTs from 3 independent experiments; Cal520 (0.1): n=12 EHTs from 3 independent experiments; Cal590 (1.0): n=5 EHTs from 2 independent experiments. B) Cal520 (0.2): n=23 EHTs from 10 independent experiments; Cal590 (1.0): n=7 EHTs from 4 independent experiments.

## Supplementary materials 2

### Incision characteristics

The EHT was implanted in a pocket underneath the epicardium, which was made using an ophthalmic knife as shown in **Supplementary Figure 2A**. During the cut, the knife was visible through the thin layer of epicardium ensuring a consistent depth each time.

Histological slides provide a macroscopic view of the implantation site, where the myocardial band is approximately ~300 µm thick and the hydrogel is clearly visible in between both myocardial layers (**Supplementary Figure 2B**). However, due to processing prior to histological staining the dimensions of the implantation site beyond the size of the myocardial flap are inaccurate. The dimensions of the hydrogel seem similar to that during implantation (~350µm), but the processing has caused the material to fold and relocate. Ku80 staining clearly shows transfer of hiPSC-CMs from the biomaterial to the myocardial flap.


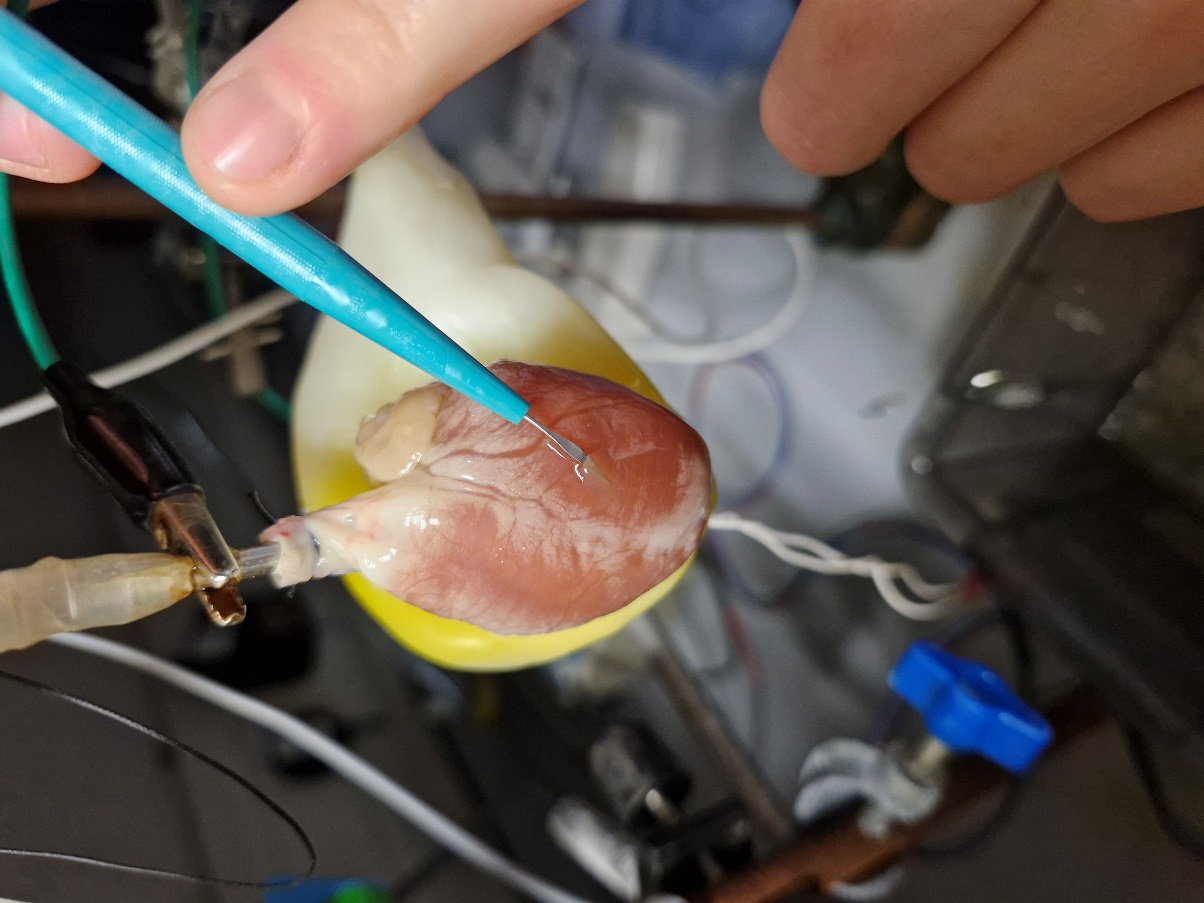

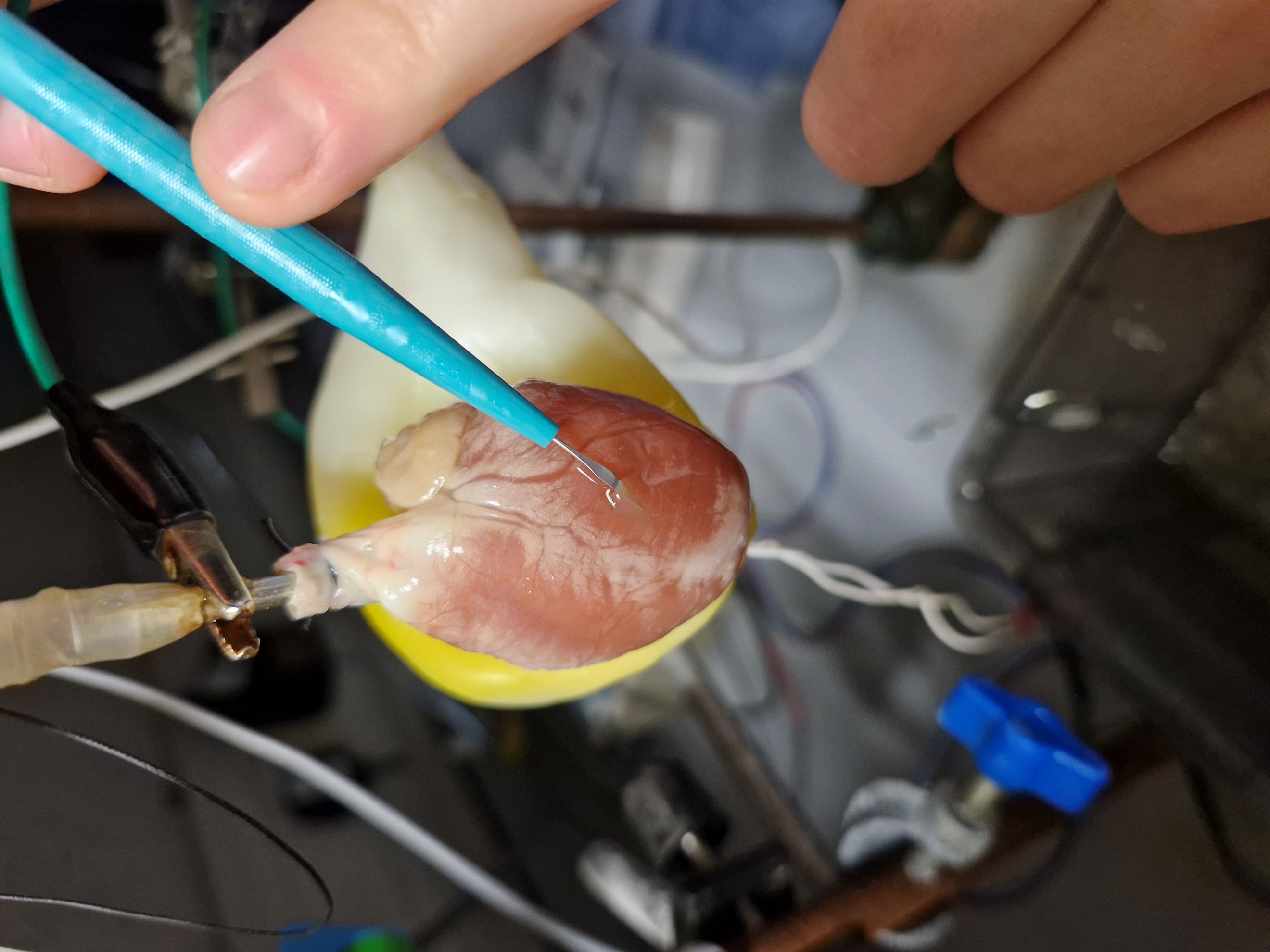


1 cm

1 cm


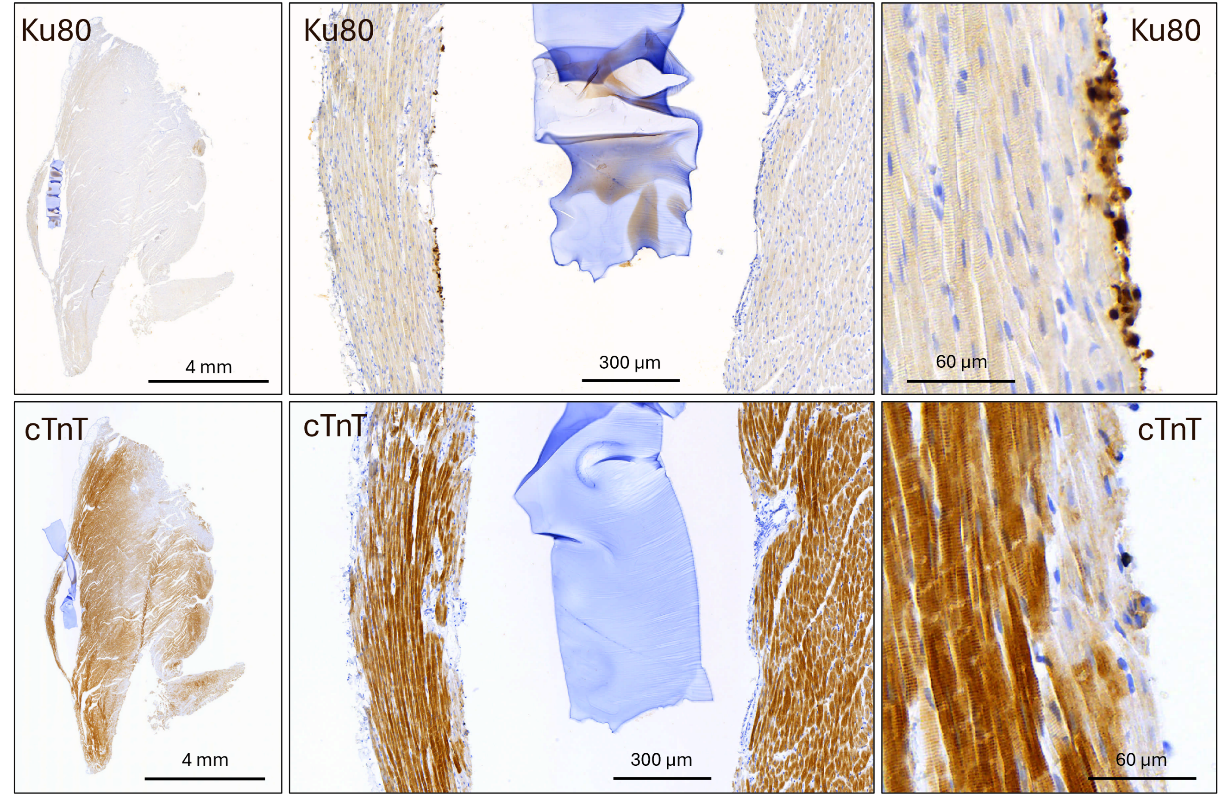


A

B

Figure 2 Incision characteristics. A) Use of an ophthalmic knife to create the superficial incision underneath the rabbit epicardium for EHT implantation. Insert: magnification of incision site showing transparency of myocardial “flap”. B) Histological preparation showing a macroscopic view of the implantation site at various magnifications. Slide is stained for Ku80 (human nuclear marker), indicating the implanted hiPSC-CMs.

## Supplementary materials 3

### Bi-domain modelling of iPSC-CM-myocardium interaction

**Supplementary Figure 3** shows a 3-D maps of voltage of two blocks of rabbit myocardium (5x5x2.5mm each) in response to the simultaneous stimulation at the points indicated. The two blocks are separated by an iPSC-CM layer of 50μm with a saline film (25-100μm). The location of the simulated voltage signal shown in **Figure 6** is indicated in the cross-section.


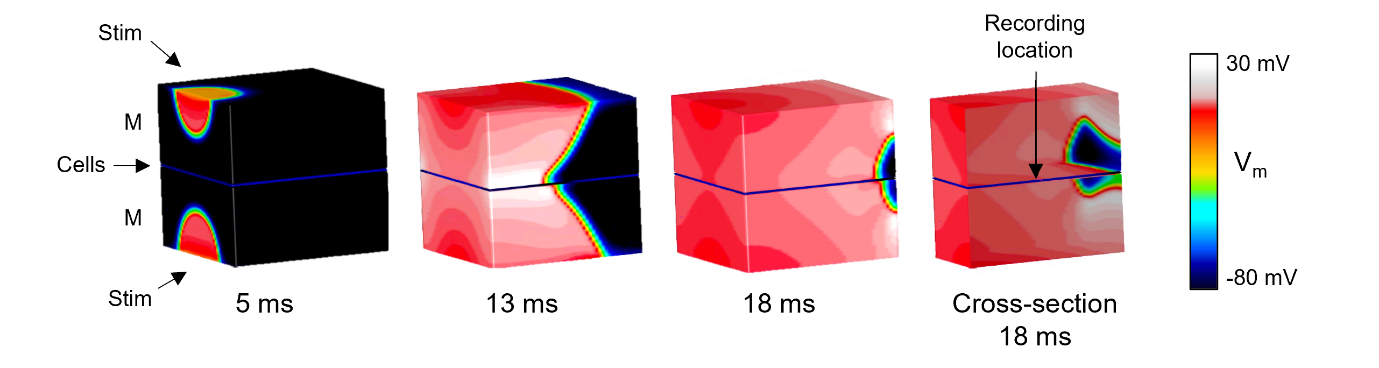


Figure 3 Example images of one run of the 3D mathematical model at three different timepoints (5, 13 and 18 ms). Stim = location where the rabbit myocardium is electrically stimulated. Cells = layer of iPSC-CMs. M = rabbit myocardium.

## Supplementary materials 4

### EHT performance over time

EHT CaTs were recorded before implantation to assess their viability and the calcium signal strength (**Supplementary Figure 4A**). Then, more recordings were made every 20-30 mins alongside the pECG of the rabbit heart, starting directly after implantation (**Supplementary Figure 4B**), for as long as possible. The traces shown in **Supplementary Figure 4C** were made approximately 60 mins post-implantation. The capability of the cells to follow various pacing frequencies (2 Hz, 2.5 Hz and 3.3 Hz) was assessed at every timepoint. The importance of the calcium signal strength is illustrated in this figure as it shows the declining calcium amplitude over time due to bleaching. Calcium trace bleaching was the main reason to record sequentially and not consistently, as sequential recording would minimize fluorescent bleaching, thus allowing to record over a longer period of time. However, this also meant potentially missing crucial data in between recordings and could therefore be seen as an important limitation of this study.


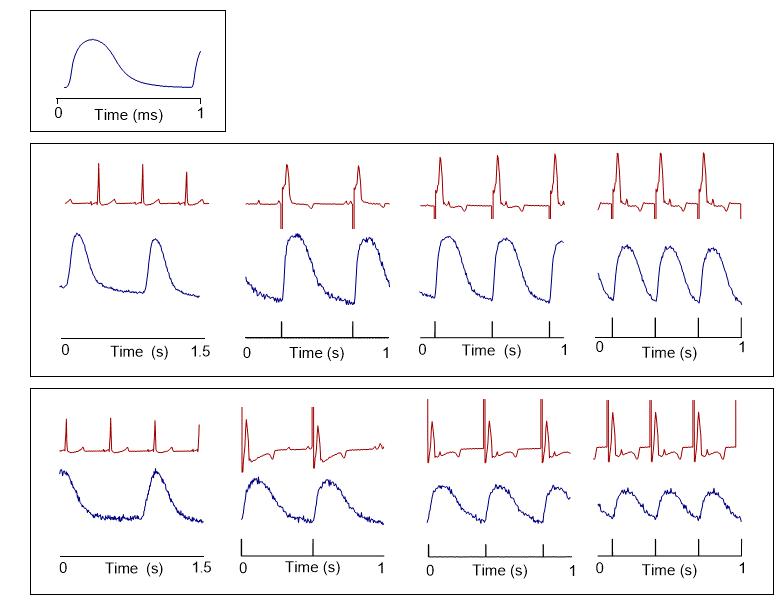


Spontaneous rate

2 Hz

2.5 Hz

3.3 Hz

A

Pre-implantation

B

C

Directly post-implantation

1hr post-implantation

Figure 4 EHT performance over time. CaTs recorded from the EHT (blue), the pECG recorded from the rabbit myocardium (red) and the electrical pacing stimulus (black). Traces were recorded before (A), directly after (B) and approximately 1 hour after implantation (C) in the absence of ventricular contraction. During recordings, EHTs and heart were paced at various pacing frequencies (2, 2.5 and 3.3 Hz) along with the spontaneous rate. All CaT recordings presented were made with the light guide. Example traces are from a non-contracting heart (N=1 heart).

## Supplementary materials 5

### Reversible contraction inhibition

To confirm the decrease and increase of LV contraction during blebbistatin incubation and wash-off, respectively, LV contraction amplitude was quantified. **Supplementary Figure 5A** shows that after 20 min of blebbistatin incubation the LV contraction was undetectable but recovered towards control levels during the wash-off phase, returning to approximately 80-90% of the original values after approx. 100 min (**Supplementary Figure 5A**). **Supplementary Figure 5B** shows the LV contraction for each recording and shows a gradual increase in LV contraction for all recordings in the Contraction Recovery group, whereas contraction remains absent in the No Contraction group. From approx. 100 min post-implantation, the motion artefacts were too large to reliably distinguish the CaT and thus the experiments were aborted from that point. Because the contraction was always uncoupled for the No Contraction group, it was possible to record for longer periods of time (**Supplementary Figure 5B**).

A

B

Figure 5 The effect of blebbistatin on LV contraction during incubation and washout. A) LV contraction in mmHg over time, recorded at 10 min intervals (N=6 hearts). Error bars represent SD. B) The symbols represent the times a CaT transient trace was sampled across all preparations and the corresponding value of the LV contraction in hearts exposed to blebbistatin continuously (no contraction group, red crosses, N=9 hearts, n=62 traces) or during a blebbistatin washout (contraction recovery group, blue plusses, N=6 hearts, n=35 traces).

## Supplementary materials 6

### Assessing potential Cal520 leak through gap junctions

Entrainment could be caused by newly formed gap junctions. To test this hypothesis, calcium transients from entrained (all traces) and non-entrained traces (5 from each group) were further analysed to assess whether the Cal520 dye has diffused through newly formed gap junctions into the rabbit cardiomyocytes where it would initiate a fluorescent calcium transient shortly after depolarization.

For this reason, data from other isolated rabbit heart experiments using calcium dyes were used to determine the time between the R-peak of the pECG and the peak of the fluorescent calcium trace (**Supplementary Materials** and **Supplementary Figure 6A**), which was 28±2 ms on average when hearts were paced at 2Hz (N=3 hearts). Then, the recorded calcium signal was averaged over the R-peak of the pECG (F’_P(Heart)_) as well as over its own upstroke (F_P(EHT)_).

Example traces are shown in **Supplementary Figure 6B.** Subsequently, the change in fluorescent signal intensity was quantified by calculating the change in fluorescent signal in diastole (F_0_) with that in systole (F_P_) using the following formula: (F_P_-F_0_)/F_0_ or ∆F/F_0_. The results are shown in **Supplementary Figure 6C** and statistical tests resulted in significant p-values when comparing ∆F’_P(Heart)_ and ∆F_P(EHT)_. Additional test to determine whether ∆F’_P(Heart)_ is significantly different from zero showed that this was not the case. Therefore, we can conclude that the Cal520 dye did not diffuse into the myocardium and thus that no gap junctions were formed. This means that gap junctions cannot be the underlying cause for the entrainment seen during this study.


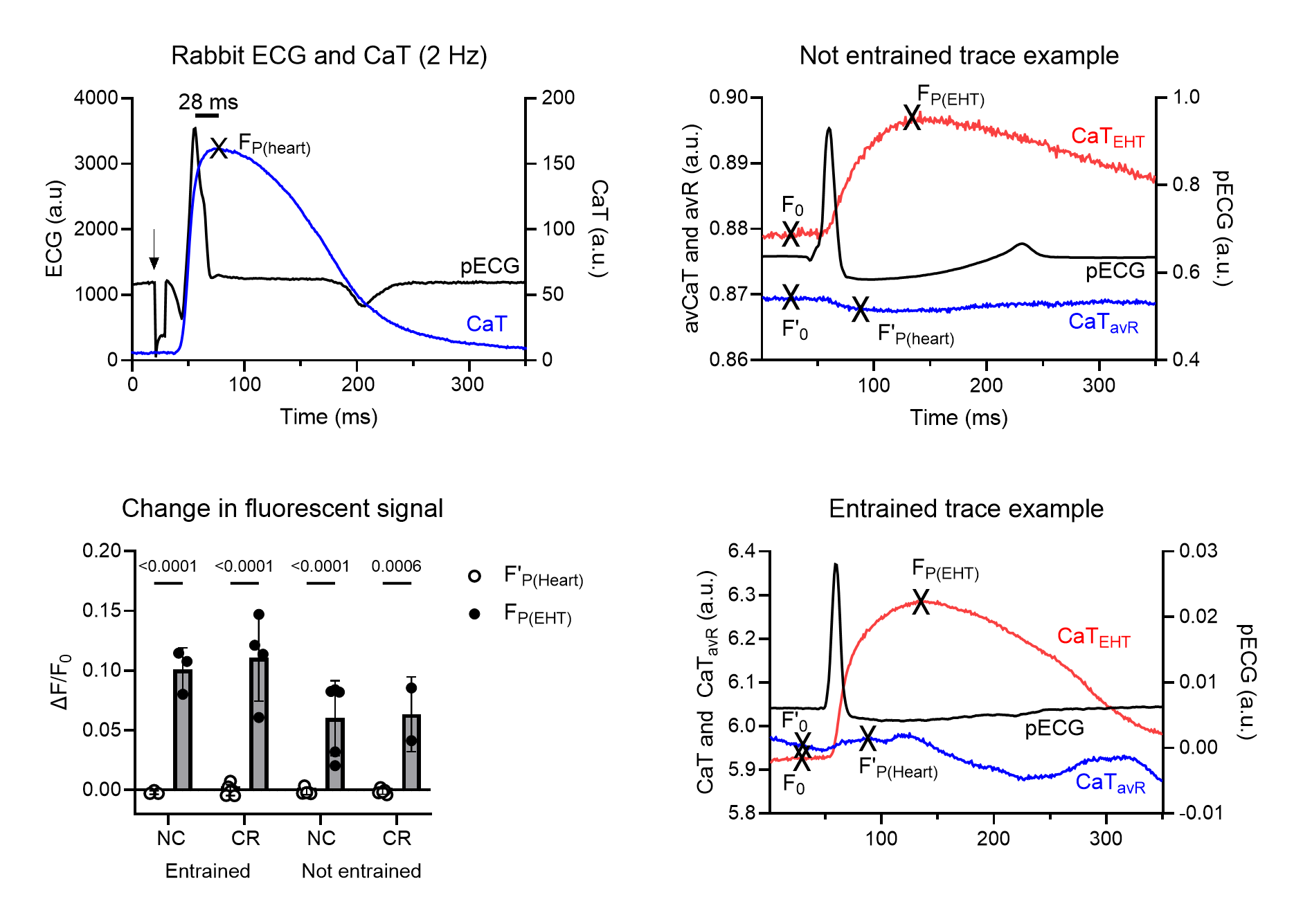


A

Bi

C

Bii

Figure 6 Assessing whether Cal520 has leaked through newly formed gap junctions between the hiPSC-CM and the rabbit myocardium. A) pECG and CaT from the LV of a rabbit heart paced at 2 Hz. The cross indicates the peak of the calcium trace taken from a representative area of the rabbit LV (F_P(Heart)_). The arrow indicates the stimulus artefact. B) Calcium traces of the EHT (CaT_EHT_, red ) along the pseudo ECG (pECG, blue) and the averaged calcium trace on the R-peak (CaT_avR_, blue) for both a non-entrained sample (i, top) and entrained sample (ii, bottom). Crosses indicate the baseline (F_0_) and peak (F_P(EHT)_) of the calcium trace of the EHT or the baseline (F’_0_) and peak (F’_P(Heart)_) of the calcium trace of the rabbit myocardium. C) Average changes in fluorescent calcium traces (∆F/F_0_) for either the EHT (grey bars (F_P(EHT)_) or the rabbit myocardium (F’_P(Heart)_). A one-way ANOVA was done to compare differences between F’_P(Heart)_ and F_P(EHT)_. A one sample T-test was performed determined that F’_P(Heart)_ values were not significantly different from zero (NC entrained [N=1 heart; n=3 traces]: p=0.1520 ; CR entrained [N=5 hearts; n=6 traces]: p=0.9760 ; NC not entrained [N=3 hearts; n=5 traces]: p=0.3610 ; CR not entrained [N=3 hearts, n=5 traces]: p=0.2831).

1. Alexander C. Mechanisms of Ventricular Arrhythmias in the Long QT Syndrome: The University of Glasgow. 2020.
